# Cupric Doping Hollow Prussian Blue Nanoplatform for Enhanced Cholesterol Depletion: a Promising Strategy for Breast Cancer Therapy and Metastasis Inhibition

**DOI:** 10.1002/advs.202409967

**Published:** 2024-11-28

**Authors:** Shuangqian Yan, Panpan Xue, Ying Sun, Tingjie Bai, Sijie Shao, Xuemei Zeng

**Affiliations:** ^1^ Key Laboratory of Microbial Pathogenesis and Interventions of Fujian Province University Biomedical Research Center of South China College of Life Sciences Fujian Normal University 1 Keji Road Fuzhou 350117 P. R. China; ^2^ The Straits Institute of Flexible Electronics (SIFE, Future Technologies) Straits Laboratory of Flexible Electronics (SLoFE) Fujian Normal University Fuzhou Fujian 350117 P. R. China; ^3^ Department of Gastroenterology Fuzhou No. 1 Hospital Affiliated with Fujian Medical University Fuzhou Fujian 350009 P. R. China

**Keywords:** cholesterol metabolism regulation, cholesterol oxidase, metal organic frameworks, tumor metabolism therapy, tumor metastasis inhibition

## Abstract

The dysregulated cholesterol metabolism in breast cancer cells drives malignancy, invasion, and metastasis, emphasizing the significance of reducing abnormal cholesterol accumulation for effective cancer treatment and metastasis inhibition. Despite its promise, cholesterol oxidase (ChOx) encounters challenge due to limited catalytic efficiency and susceptibility to harsh conditions. To overcome these hurdles, biocompatible nanoplatforms (Cu‐HPB/C) tailored for efficient cholesterol depletion are introduced. Cu^2+^‐doped hollow Prussian blue (Cu‐HPB) acts as a carrier, shelter, and enhancer for ChOx, bolstering tumor‐targeting ability, stability, and enzymatic activity. Tumor‐responsive released Cu^2+^ notably augments ChOx activity, facilitating cholesterol depletion and disrupting lipid rafts, thereby impeding cell invasion and migration. Additionally, H_2_O_2_ generated from the oxidase reaction enhances Cu‐HPB's chemo dynamic therapeutic efficacy. Transcriptomic analysis validates Cu‐HPB/C's impact on cholesterol homeostasis and reveals cell death mechanisms including oxidative stress, ferroptosis, cuproptosis, and apoptosis. Demonstrating therapeutic efficacy in both 4T1 tumor subcutaneous and metastasis mouse models, the study presents a direct and effective strategy for tumor therapy and metastasis inhibition through enhanced cholesterol depletion.

## Graphical abstract

This study introduces a Cu^2+^‐doped hollow Prussian blue materials concerted with cholesterol oxidase (ChOx), termed Cu‐HPB/C, engineered for tumor‐targeting, in vivo enzymatic stabilization and boosting of ChOx activity, aiming to initiate tumor therapy and metastasis inhibition by enhanced cholesterol depletion. The findings underscore the crucial role of cholesterol metabolism homeostasis in suppressing cancer metastasis.

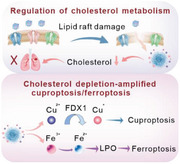

## 1 Introduction

Emerging evidence highlights the crucial role of altered metabolism in sustaining tumor proliferation and metastasis, particularly through increased demands for nutrients such as glucose, pyruvate, cholesterol, and fatty acids.^[^ ^1^, ^2^, ^3^ ^]^ This metabolic flexibility drives tumor invasion and metastasis, making metabolism an attractive target for combating primary tumors and refractory metastasis.^[^ ^4^, ^5^, ^6^ ^]^ Hyperactive cholesterol metabolism, observed in metastasizing prostate, lung, and breast cancers, is linked to cell migration and invasion.^[^ ^7^, ^8^, ^9^ ^]^ Cholesterol plays key roles in lipid raft formation,^[^ ^10^ ^]^ membrane biogenesis,^[^ ^11^, ^12^ ^]^ cancer progression,^[^ ^13^, ^14^ ^]^ and immune cell function.^[^ ^15^, ^16^ ^]^ It activates canonical Wnt signaling pathways to facilitate cell proliferation and differentiation, while cholesterol in lipid rafts can activate the RAS‐MAPK signaling pathway, inducing cell migration through the epithelial‐mesenchymal transition process.^[^ ^5^, ^17^ ^]^ Additionally, the primary cholesterol metabolite, 27‐hydroxycholesterol, enhances the tumorigenic and metastatic capacity of tumor cells.^[^ ^18^, ^19^ ^]^ Therefore, inhibiting cholesterol metabolism or blocking its synthesis holds substantial potential for metastasis inhibition.

Several studies have successfully targeted cholesterol metabolism to treat cancer.^[^ ^20^, ^21^, ^22^, ^23^ ^]^ For instance, using the cholesterol synthesis inhibitor simvastatin, researchers developed a nanoplatform to block cholesterol synthesis and reverse the immunosuppressive tumor microenvironment in cholesterol‐abundant oral squamous cell carcinoma, improving photo‐immunotherapy efficacy.^[^ ^24^ ^]^ Additionally, addressing the overexpression of squalene epoxidase (SQLE) in oral squamous cell carcinoma with the SQLE inhibitor terbinafine reversed the tumor microenvironment and enhanced immunotherapy by blocking cholesterol synthesis.^[^ ^25^ ^]^ However, tumor cells can acquire cholesterol from various sources,^[^ ^26^ ^]^ limiting the efficacy of cholesterol synthesis inhibitors.

Cholesterol oxidase (ChOx) presents a potential solution by depleting both de novo synthesized and dietary cholesterol, thereby reducing total cholesterol content. Recent advancements include modifying hollow organic frameworks (HOFs) with ChOx and LXR‐623 to decrease cholesterol uptake and increase its efflux, achieving antibody non‐dependent immunotherapy tailored for glioblastoma.^[^ ^27^ ^]^ Additionally, constructing Hf‐TBP/COD to deplete membrane cholesterol has been shown to enhance T cell reinvigoration via pyroptosis.^[^ ^28^ ^]^ Our group designed Fe‐MOF nanozymes concerted with ChOx to initiate cholesterol depletion‐mediated ferroptosis and immunotherapy.^[^ ^29^ ^]^ Despite these advancements, it remains unclear how cholesterol metabolism impacts metastasis inhibition independently of the immune system. Moreover, ChOx's fragility and vulnerability to harsh physicochemical environments restrict its broader bioengineering applications.^[^ ^30^, ^31^ ^]^

Various strategies and functional materials have recently been developed to advance innovative cancer therapies.^[^ ^32^, ^33^, ^34^ ^]^ Among them, Metal‐organic frameworks, particularly Prussian blue (PB), hold promise in drug delivery systems due to their biocompatibility, large surface area, tunable porosity, and potential to encapsulate enzymes.^[^ ^35^, ^36^, ^37^, ^38^, ^39^ ^]^ Previous evidence suggests that Cu^2+^ can activate ChOx and enhance its enzyme activity.^[^ ^40^ ^]^ Motivated by these findings, we hypothesized that Cu^2+^‐doped hollow PB (Cu‐HPB) could be an optimal candidate for achieving high loading capacity, effective delivery, and protection and enhancement of encapsulated ChOx enzyme activity.

**Figure 1:**
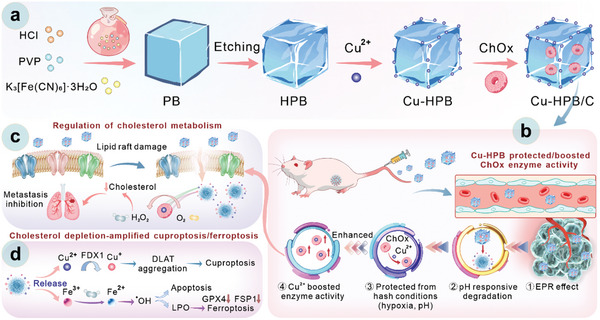
Synthesis and tumor inhibition mechanisms of Cu‐HPB/C nanoplatforms. a) Diagram illustrating the synthetic processes of Cu‐HPB/C. b) Schematic depicting tumor targeting, degradation, ChOx protection, and ChOx activity enhancement by Cu‐HPB in tumor‐bearing mouse models. c,d) Schematic illustrations of Cu‐HPB/C's roles in modulating cholesterol metabolism and inhibiting metastasis (c) and augmenting cuproptosis/ferroptosis (d).

Herein, we report our findings on Cu‐HPB/C nanoplatforms for cholesterol depletion‐mediated triple‐negative breast cancer therapy and metastasis inhibition (**Figure** **1**). Cu‐HPB acts as a carrier, sanctuary, and enhancer for ChOx, providing ChOx with tumor‐targeting ability, stability, and in vivo enzyme activity boosting for the first time. The acidity‐triggered release of Cu^2+^ enhances ChOx activity, facilitating effective cholesterol depletion and H_2_O_2_ generation. Cholesterol depletion disrupts cholesterol homeostasis and lipid raft function, ultimately preventing tumor invasion and metastasis. Furthermore, the generated H_2_O_2_ enhances the antitumor effects of Cu‐HPB‐mediated chemodynamic (CDT), ferroptosis, and cuproptosis. Our fabricated Cu‐HPB/C nanoparticles demonstrate robust antitumor and antimetastatic effects in 4T1 tumor subcutaneous and metastasis mouse models.

## 2 Results and Discussion

### 2.1 Characterization and Optimization of Cu‐HPB

**Figure 2:**
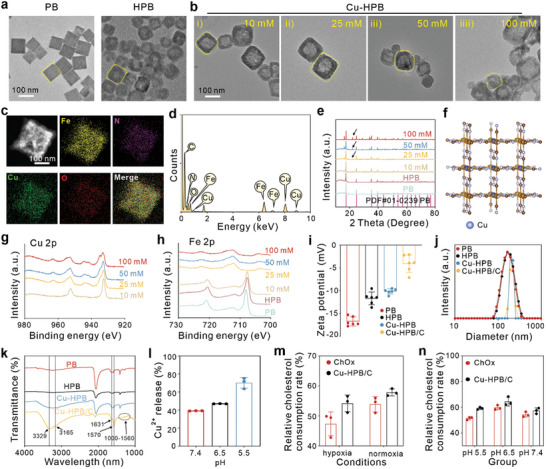
Characterization of Cu‐HPB/C and its protection/boosting capacity to ChOx. a) TEM images of PB and HPB. b) TEM images of Cu‐HPB after mixing with various concentrations of CuSO_4_. Scale bars are 100 nm. c,d) Elemental mapping images (c) and EDX analysis of (d) Cu‐HPB after mixing with 50 mM CuSO_4_ for 8 h at room temperature. e) XRD spectra of PB, HPB, and Cu‐HPB after reacting with various concentrations of CuSO_4_. f) Simplified geometrical structure of Cu‐HPB. g,h) XPS spectra of Cu 2p peak (g) and Fe 2p peak (h) from Cu‐HPB. i–k) Zeta potentials (i) (n = 6), hydrodynamic sizes (j), and FTIR spectra (k) of Cu‐HPB/C during various modifications. l) Cu^2+^ release behaviors of Cu‐HPB/C under various pH values (n = 3). m,n) Relative cholesterol consumption rate of Cu‐HPB/C and free ChOx under hypoxic (m) and acidic (n) conditions(n = 3).

**Figure 3:**
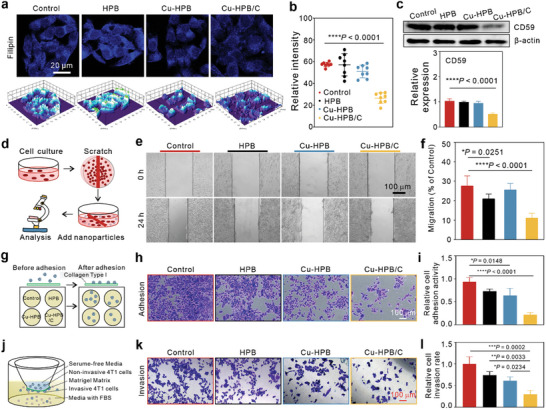
Cholesterol depletion, and antimetastasis effect of Cu‐HPB/C in vitro. a,b) CLSM images (a) and corresponding quantitative data (b) of Filipin‐stained 4T1 cells after treatments (n = 8). Scale bars: 20 µm. c) Western blot analysis of CD59 expression in 4T1 cells with indicated treatments (n = 3). d–f) Schematic representation (d), representative bright field images (e), and the migration rate (f) of 4T1 cells treated with different formulations (n = 3). g–i) Schematic illustration (g), representative staining filed images (h), and relative adhesion activity (i) of 4T1 cells after treatments (n = 3). j–l) Schematic representation (j), representative staining filed images (k), and relative invasion rate (l) of 4T1 cells after treatments (n = 3). Statistical significance denoted as ^*^ *p* < 0.05, ^**^ *p* < 0.01, ^***^ *p* < 0.001, ^****^ *p* < 0.0001, analyzed by one‐way ANOVA, followed by Dunnett's multiple comparisons test. Data represent mean ± standard deviation (s.d.).

We synthesized PB through self‐assembly at a high temperature followed by acidic etching to obtain HPB nanoparticles.^[^ ^41^ ^]^ Transmission electron microscopy (TEM) images revealed that PB nanoparticles exhibit a cuboidal morphology and high monodispersity, while HPB possesses an apparent hollow mesoporous structure (**Figure** **2**a). The synthesized PB nanoparticles exhibited pH‐responsive degradation, as confirmed by TEM imaging. As shown in Figure S1 (Supporting Information), the PB maintained its structural integrity after 12 and 24 h in PBS at pH 7.4. In contrast, significant collapse and complete disintegration were observed after the same time periods in PBS at pH 5.5, confirming the pH‐responsive degradation of PB and facilitating iron ion release under acidic conditions. Subsequently, copper ions were doped into HPB via cation exchange. HPB nanoparticles were stirred with various concentrations of CuSO_4_ (10, 25, 50, and 100 mM) at room temperature for 8 h, and the morphologies of Cu‐HPB were visualized. As shown in Figure 2b, low concentrations (10, 25, and 50 mM) of Cu^2+^ did not influence the original morphology of HPB. In contrast, HPB exhibited a deficiency in its original cuboidal morphology after mixing with a high concentration of 100 mM Cu^2+^, which is attributed to lattice distortion and interactomics coupling effects changes induced by abundant Cu^2+^ doping.^[^ ^42^ ^]^ Afterward, the influence of Cu^2+^ doping on the crystal structure and element distribution of Cu‐HPB was systematically investigated. The element mapping results illustrated the uniform distribution of Fe and Cu elements in Cu‐HPB after reacting with 50 mM CuSO_4_ (Figure 2c). Energy dispersive X‐ray (EDX) detection further indicated the successful Cu doping in HPB (Figure 2d). X‐ray diffraction (XRD) was performed to characterize the crystal structure of all samples, and the results showed peaks consistent with the stimulated ones, validating their successful synthesis (Figure 2e). Additionally, new characteristic peaks appeared in Cu‐HPB with elevated Cu^2+^ mixing (25, 50, and 100 mm), indicating Cu^2+^ is chemical bonding with [Fe(CN)_6_] and covalent binding to the cyanide ligands.^[^ ^43^ ^]^ A simplified geometrical structure of Cu‐HPB is illustrated in Figure 2f. Next, X‐ray photoelectron spectroscopy (XPS) was utilized to investigate the element distribution in Cu‐HPB. The results indicated that both PB and HPB consist of Fe, C, N, and O elements (Figure S2, Supporting Information). After Cu^2+^ doping, an obvious Cu 2p peak was detected in Cu‐HPB, verifying successful Cu^2+^ doping (Figure 2g; FigureS3, Supporting Information). In addition, the Fe 2p peak after Cu^2+^ doping gradually shifted to the right (Figure 2h), indicating an increase in the Cu/Fe ratio. The phenomenon was attributed to electron transfer from Cu^+^ to Fe^3+^, further supported by the leftward shift of the Cu 2p peak, which verifies this mechanism.^[^ ^44^, ^45^ ^]^ We selected 50 mM Cu^2+^‐exchanged Cu‐HPB for subsequent experiments due to its high Cu^2+^ ratio and intact morphology. As evidenced by the o‐phenylenediamine (OPD) chromogenic reaction, we found that Cu‐HPB exhibited superior catalytic activities to H_2_O_2_ compared to HPB (Figure S4, Supporting Information), indicating its peroxidase (POD)‐like activity and showed potential in tumor‐specific CDT.

### 2.2 Characterization of Cu‐HPB/C

Cu‐HPB/C was fabricated by simply mixing ChOx and Cu‐HPB in a buffer solution. ChOx can be loaded into the platform's internal cavity and porous matrix via pore loading and confinement effects, respectively.^[^ ^46^ ^]^ However, non‐specific binding of ChOx to Cu‐HPB may also occur, resulting in surface absorption.^[^ ^47^ ^]^ The successful fabrication of Cu‐HPB/C was systematically characterized. The emergence of absorption between 300–600 nm in UV–vis spectra of Cu‐HPB was displayed after ChOx loading, verifying successful loading in the Cu‐HPB (Figure S5, Supporting Information). The BCA Protein Assay Kit was used to measure the ChOx loading rate, which was determined to be 86.75% as depicted in Figure S6 (Supporting Information). Zeta potentials of PB, HPB, Cu‐HPB, and Cu‐HPB/C were −16.8, −11.5, −10.3, and −3.9 mV, respectively (Figure 2i). The negative potential of PB is attributed to negatively charged organic linkers and absorbent polyvinyl pyrrolidone molecules during synthesis.^[^ ^48^ ^]^ HPB and Cu‐HPB exhibited elevated potentials compared to PB, possibly due to acid etching and Cu^2+^ ion exchange exposing cation ions on the surface of PB. Interestingly, ChOx loading elevated the zeta potential of Cu‐HPB, which may result from that ChOx was also absorbed on the surface of the Cu‐HPB, replacing the highly negatively charged polyvinyl pyrrolidone molecules.^[^ ^28^ ^]^ As shown in Figure 2j, the hydrodynamic size of Cu‐HPB/C was 220 nm. Fourier transform infrared (FTIR) spectroscopy of Cu‐HPB/C displayed an absorption peak at 3329, 3165, 1631, and 1570 cm^−1^ compared to Cu‐HPB without ChOx modification. The peak located at 3329 and 3165 cm^−1^ corresponded to the C─H stretching of ChOx, while the peak located at 1631 and 1570 cm^−1^ corresponded to the C═O, CN, and NH stretching of amide bands of ChOx (Figure 2k). Zeta potential values and hydrodynamic size of Cu‐HPB/C dissolved in PBS and FBS remained stable within 7 days of monitoring, validating satisfactory stability (Figure S7, Supporting Information). Taken together, these results strongly demonstrate the successful construction of Cu‐HPB/C.

### 2.3 Protections and Enhancement of ChOx Activity by Cu‐HPB/C

Previous studies have indicated that Cu^2+^ acts as an activator to enhance ChOx activity.^[^ ^40^ ^]^ We measured ChOx enzyme activity at various concentrations of Cu^2+^ using a 4‐amino‐antipyrine colorimetric method to verify its impact.^[^ ^49^ ^]^ The results showed a gradual increase in ChOx activity with increasing Cu^2+^ concentrations, reaching a 2‐fold increase at a concentration of 0.2 µM Cu^2+^, indicating that even relatively low concentrations of Cu^2+^ can boost ChOx activity (Figure S8, Supporting Information). The pH‐responsive release of iron and copper ions was further investigated. As depicted in Figure S9 (Supporting Information), Cu‐HPB/C showed maximal iron ions release in acidic conditions, a prerequisite for inducing ferroptosis in vitro. Additionally, Cu‐HPB/C exhibited significant Cu^2+^ release under acidic conditions (Figure 2l), ensuring its ability to enhance ChOx activity in the acidic tumor microenvironment. Next, the protective ability of Cu‐HPB for ChOx was evaluated. Under normoxia conditions, both ChOx and Cu‐HPB/C possessed considerably higher rates of cholesterol consumption at 53.86% and 57.82%, respectively, implying executable ChOx enzyme activity. The increased rate of cholesterol consumption by Cu‐HPB/C was attributed to the enhanced capacity of Cu^2+^ to activate ChOx. Under hypoxia conditions, ChOx exhibited a low cholesterol consumption rate of 47.36% (Figure 2m). Upon loading into Cu‐HPB, Cu‐HPB/C displayed an increased rate of 54.27%. ChOx exhibited enhanced activity in moderately acidic conditions but showed a marked decrease at pH 5.5 (Figure S10, Supporting Information). The cholesterol consumption rate of ChOx declined significantly as pH shifted from 7.4 to 6.5 and 5.5, whereas Cu‐HPB/C maintained a consistent rate across these pH levels (Figure 2n; Figure S11, Supporting Information), indicating that Cu‐HPB provides both protection and enhancement of ChOx activity in acidic and hypoxic environments, demonstrating the protective and enhancing effect of Cu‐HPB on ChOx activity under hypoxic and acidic conditions.

### 2.4 Cellular Uptake Pathway of Cu‐HPB/C

Cu‐HPB/C was labeled with fluorescein isothiocyanate (FITC) to visualize its cellular uptake behavior in 4T1 cells. Confocal images in Figure S12 (Supporting Information) illustrate the bright green fluorescent intensity concentrated in cytoplasm over time, indicating successful Cu‐HPB/C uptake by 4T1 cells. Increasing mean fluorescence intensity (MFI) in flow cytometric results further confirmed the efficient uptake of Cu‐HPB/C (Figure S13, Supporting Information). Subsequently, the cellular uptake mechanisms of Cu‐HPB/C were systematically investigated.^[^ ^50^, ^51^ ^]^ The results, shown in Figure S14 (Supporting Information), indicated that cells treated with low temperature (4 °C) (energy‐dependent pathway), β‐CD (caveolin‐dependent endocytosis inhibitor), cytochalasin D (micropinocytosis inhibitor), and chlorpromazine (clathrin‐dependent endocytosis inhibitor), exhibited inhibited Cu‐HPB/C uptake, suggesting the involvement of all the aforementioned pathways in cellular uptake. Cells treated with wortmannin (phagocytosis and macropinocytosis inhibitor) exhibited slight inhibition, indicating that phagocytosis and macropinocytosis did not primarily participate in the cellular uptake of Cu‐HPB/C. In addition, the Cu‐HPB/C demonstrated lysosomal escape capacities, as confirmed by a co‐localization fluorescent assay. As shown in Figure S15 (Supporting Information), Cu‐HPB/C was significantly localized in lysosomes at 2 h post‐incubation (Pearson coefficient (PC) = 0.59), with increased accumulation observed at 6 h (PC = 0.63). By 10 h, only minimal Cu‐HPB/C remained within the lysosome (PC = 0.41), indicating successful lysosomal escape.

### 2.5 Intracellular Cholesterol Depletion

After confirming the successful uptake of Cu‐HPB/C by 4T1 cells, it's in vitro cholesterol depletion ability was visualized using Filipin staining, a fluorescent dye that specifically binds to cholesterol. 4T1 cells were treated with different nanoparticles and imaged using confocal laser scanning microscopy (CLSM) (**Figure** **3**a). Compared to the control group, cells treated with HPB and Cu‐HPB showed minimal changes in blue fluorescence, whereas those treated with Cu‐HPB/C exhibited reduced fluorescence (Figure 3b), demonstrating its excellent ability to deplete cholesterol in cancer cells.

### 2.6 Lipid Raft Destruction

Lipid rafts are enriched with cholesterol and play a vital role in various cellular functions such as adhesion, motility, and signal transduction.^[^ ^52^ ^]^ To assess the impact of cholesterol depletion on lipid raft function, we evaluated the expression of raft‐specific membrane protein CD59 (Figure 3c). Western blotting results indicated that HPB and Cu‐HPB treatments did not alter the protein expression levels of CD59. However, following ChOx loading, Cu‐HPB/C treatments significantly down‐regulated CD59 expression, suggesting dysfunction of lipid rafts occurred following cholesterol depletion.

### 2.7 Cu‐HPB/C‐Mediated Inhibition of Adhesion, Migration, and Invasion

Previous studies have reported that hyperactive cholesterol metabolism in breast cancer cells contributes to cell malignancy, invasion, and metastasis.^[^ ^7^, ^53^, ^54^ ^]^ Inspired by the remarkable cholesterol depletion and disruption of lipid rafts induced by Cu‐HPB/C, we hypothesized that cholesterol depletion could effectively inhibit cell adhesion, migration, and invasion. Wound‐healing assays (Figures 3d–f) and cell‐matrix adhesion assays (Figures 3g–i) were conducted to assess cell migration and adhesion capabilities. HPB and Cu‐HPB treatments resulted in a partial reduction in cell adhesion, whereas Cu‐HPB/C treatments led to a reduction of more than 78.07% in cell adhesion, along with a smaller wound‐healing area (reduction of 16.41%), confirming the effective inhibition of adhesion and migration by Cu‐HPB/C. Additionally, transwell invasion assay results (Figures 3j–l) were performed to verify the reduced migratory capacity of Cu‐HPB/C (invasion rate decreased to 29.93%). In conclusion, those results suggest the promising potential of Cu‐HPB/C in antimetastasis.

### 2.8 In Vitro Therapy by Cu‐HPB/C

**Figure 4:**
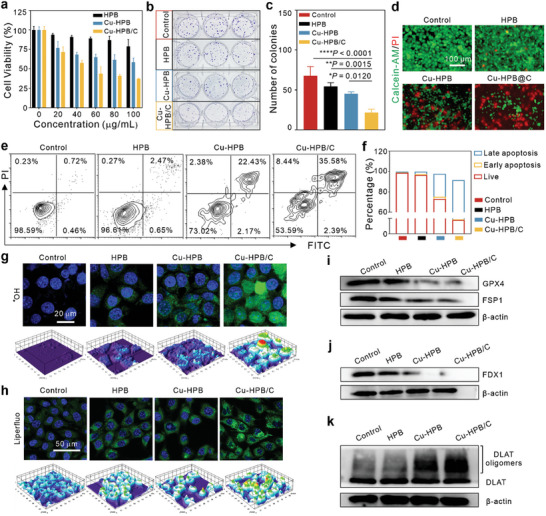
In vitro therapy by Cu‐HPB/C. a) Cell viability of 4T1 cells under different treatments (n = 5). b,c) Cell colony formation images (b) and corresponding statistical results (c) of 4T1 cells treated with indicated treatments (n = 3). d) Calcein AM/PI‐staining of 4T1 cells using different formulations. Scale bars: 100 µm. e,f) Flow cytometric analysis (e) and quantitative assessment (f) of apoptosis rates in 4T1 cells following treatment (n = 3). g) Intracellular ^•^OH levels of 4T1 cells post‐treatment. Scale bars: 20 µm. h) Intracellular LPO levels of 4T1 cells after treatment. Scale bars: 50 µm. i) Western blot analysis of GPX4 and FSP1 expression. j) Western blot analysis of FDX1 expression in 4T1 cells after treatment. k) Expression levels of DLAT and its oligomers. Statistical significance is denoted as ^*^ *p* < 0.05, ^**^ *p* < 0.01, ^****^ *p* < 0.0001, analyzed by one‐way ANOVA, followed by Dunnett's multiple comparisons test. Data represent mean ± s.d.

Subsequently, we assessed the anti‐tumor efficacy of Cu/HPB/C through a CCK‐8 assay in breast cancer cells. As illustrated in **Figure** **4**a, HPB exerted minimal effects on the viability of 4T1 cells, while Cu‐HPB demonstrated concentration‐dependent toxicity, likely attributed to its heightened H_2_O_2_‐catalytic activity compared to HPB. In contrast, Cu‐HPB/C significantly decreased the cell viability in a concentration‐dependent manner. Moreover, Cu‐HPB/C notably inhibited cell colony formation compared to other groups (Figures 4b,c). Cell live/dead staining revealed that Cu‐HPB/C exhibited superior cell‐killing ability compared to Cu‐HBP (Figure 4d), further demonstrating that ChOx enhances the therapeutic efficacy of Cu‐HBP.

We then investigated the cell death mechanisms induced by Cu‐HPB/C. As shown in apoptosis analysis (Figures 4e,f), HPB had negligible influence on cells, while Cu‐HPB induced a 26.98% percentage of cell apoptosis. In comparison, Cu‐HPB/C‐treated cells exhibited ≈46.41% apoptosis rate, suggesting that ChOx enhances cell apoptosis induced by Cu/HPB. However, apoptosis alone does not fully account for cell death. Considering the presence of Fe^3+/2+^ and Cu^2+^ in Cu‐HPB, which may induce cell ferroptosis and cuproptosis, respectively, we explored the roles of these mechanisms in cell death. Elevated cellular hydroxyl radical (^•^OH) levels have been shown to induce cell apoptosis and ferroptosis.

Therefore, ^•^OH levels in 4T1 cells following treatment were evaluated by BBoxiProbeO26 fluorescence staining. As depicted in Figure 4g, fluorescence signals were higher in cells treated with Cu‐HPB than those treated with HPB. Cu‐HPB/C significantly induced ^•^OH generation in cells. Similar results were observed with Liperfluo staining of lipid peroxides (LPOs), an indicator of ferroptosis (Figure 4h). These findings suggest that Cu‐HPB/C promotes cellular ^•^OH generation and induces cell ferroptosis. Furthermore, Cu‐HPB/C significantly downregulated the levels of glutathione peroxidase 4 (GPX4) and ferroptosis suppressor protein 1 (FSP1) in cells compared to the Cu‐HPB group (Figure 4i; Figure S16, Supporting Information). This could be attributed to ChOx consuming cholesterol and disrupting lipid rafts, which inhibits the outputs of GPX4 and FSP1. Additionally, we found that Cu‐HPB reduced the expression of ferredoxin‐1 (FDX1), and ChOx loading enhanced its inhibition efficiency (Figure 4j; Figure S17, Supporting Information). Moreover, Cu‐HPB/C significantly induced oligomerization of dihydrolipoamide S‐acetyltransferase (DLAT) (Figure 4k), validating cellular cuproptosis. Taken together, the above results demonstrate that Cu‐HPB/C induces cell death through a synergistic manner involving apoptosis, ferroptosis, and cuproptosis.

### 2.9 Therapeutic Mechanism of Cu‐HPB/C

**Figure 5:**
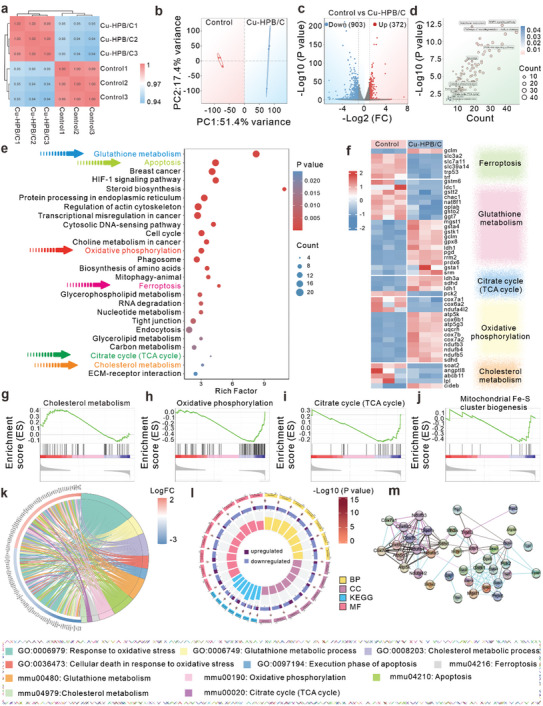
Transcriptomic sequencing analysis therapeutic mechanisms of Cu‐HPB/C in 4T1 cells. a) Heat map illustrating the correlation between samples (n = 3). b) PCA of transcriptomic sequencing data between the Control (PBS) and Cu‐HPB/C groups. c) Volcano plots comparing all gene expressions between the Control and Cu‐HPB/C groups. Blue: downregulated DEGs; Red: upregulated DEGs. d) Enrichment analysis of metabolic pathways. e) KEGG enrichment of pathways responsive to Cu‐HPB/C. f) Heat map depicting the differential expression of genes related to ferroptosis, GSH metabolism, the TCA cycle, oxidative phosphorylation, and cholesterol embolism. g–j) GSEA enrichment analysis of cholesterol embolism (g), oxidative phosphorylation (h), the TCA cycle (i), and mitochondrial Fe‐S cluster protein biogenesis (j). k) Chordal plots of KEGG analysis. l) Circos plots of KEGG and GO analysis. m) Functional association networks of proteins depicted in f.

To elucidate the therapeutic mechanism of Cu‐HPB/C, we compared the differentially expressed genes (DEGs) through transcriptomic sequencing of 4T1 cancer cells treated with control (PBS) and Cu‐HPB/C. Correlation analysis demonstrates high comparability between the two groups (**Figure** **5**a; Figure S18, Supporting Information). Principal component analysis (PCA) revealed a distinct separation between the control and Cu‐HPB/C groups, indicating significant differences in their DEGs (Figure 5b). Specifically, Cu‐HPB/C‐treated cells exhibited substantial changes in gene expression (Figure S19, Supporting Information), with 903 downregulated and 372 upregulated genes (Figure 5c). Enrichment analysis of DEGs indicated that Cu‐HPB/C affected several cellular pathways, including cholesterol metabolism, the citrate cycle (TCA cycle), ferroptosis, oxidative phosphorylation, apoptosis, PI3K‐Akt signaling, and GSH metabolism (Figure 5d). Kyoto Encyclopedia of Genes and Genomes (KEGG) enrichment analysis further confirmed that Cu‐HPB/C modulated genes associated with various cellular metabolic pathways, such as cholesterol metabolism, the TCA cycle, GSH metabolism, carbon metabolism, and biosynthesis of amino acids (Figure 5e). Additionally, Cu‐HPB/C influenced cellular survival pathways, including apoptosis, cell cycle, and ferroptosis. Figure 5f provides a detailed view of DEGs related to ferroptosis, GSH metabolism, the TCA cycle, oxidative phosphorylation, and cholesterol metabolism. Cu‐HPB/C decreased the system Xc^−^ genes, SLC3A2 and SLC7A11, which are crucial for cellular cystine uptake and subsequent GSH synthesis. As GSH is both an antioxidative substrate and a cofactor of GPX4, its depletion hampers oxidative stress defense and promotes ferroptosis. Thus, Cu‐HPB/C elevates cellular ROS levels and activates ferroptosis through system Xc^−^ inhibition.

Gene set enrichment analysis (GSEA) revealed a negative correlation with cholesterol metabolism, indicating that Cu‐HPB/C disrupts cholesterol metabolism (Figure 5g). Positive correlations with oxidative phosphorylation (Figure 5h) and the TCA cycle (Figure 5i) suggest that Cu‐HPB/C significantly impacts cellular oxidative stress, promoting cell death. In cancer cells, the overload Cu^2+^ from Cu‐HPB/C is reduced to Cu^+^ by FDX1, leading to Fe‐S cluster protein loss and elevated proteotoxic stress, culminating in cuproptosis. Figure 5j shows that Cu‐HPB/C promotes the biogenesis of mitochondrial Fe‐S clusters, likely as a feedback response to the degradation of Fe‐S clusters induced by cuproptosis. Additionally, Chordal (Figure 5k) and Circos (Figure 5l; Figure S20, Supporting Information) plots from KEGG and Gene Ontology (GO) enrichment analyses highlighted notable enrichment in metabolism and cell death pathways, including cholesterol metabolism, GSH metabolism, response to oxidative stress, the TCA cycle, oxidative phosphorylation, apoptosis, and ferroptosis. The related protein interactions are depicted in Figure 5m. These findings suggest that Cu‐HPB/C induces cell death through modulating cholesterol and GSH metabolisms, oxidative stress, apoptosis, ferroptosis, and cuproptosis.

### 2.10 Biosafety Evaluation

**Figure 6:**
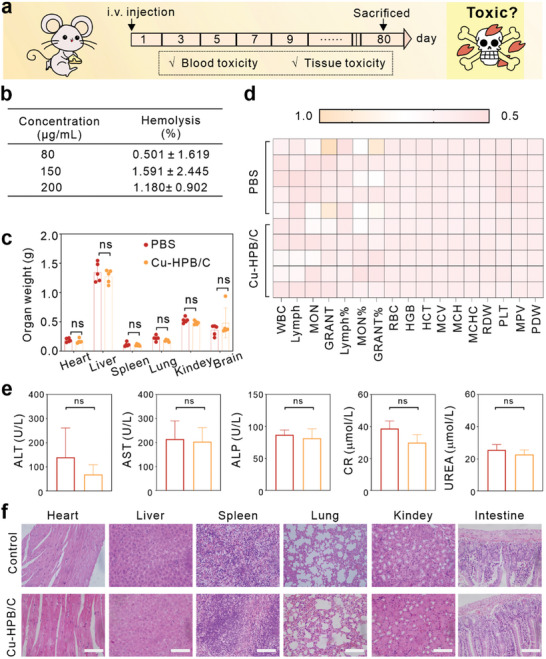
Biosafety evaluation (n = 5). a) Treatment schedule. b) Hemolysis ratio of red blood cells at different concentrations of Cu‐HPB/C. c–e) Organ weights (c), hematological analysis (d), and serum biochemical analysis of (e) from mice treated on day 80. f) H&E staining of organs. Scale bar: 100 µm. Statistical significance denoted as ns: not significant, analyzed by one‐way ANOVA, followed by Dunnett's multiple comparisons test. Data represent mean ± s.d.

Next, we assessed the long‐term biosafety and biocompatibility of Cu‐HPB/C (**Figure** **6**a). The hemolysis rate of red blood cells did not exceed 5% even at Cu‐HPB/C concentrations as high as 200 µg mL^−1^ (Figure 6b; Figure S21, Supporting Information). Furthermore, Cu‐HPB/C induced minimal schistocytes in vivo (Figure S22, Supporting Information), confirming its excellent biocompatibility. Subsequently, healthy Balb/c mice received either PBS or 20 mg kg^−1^ of Cu‐HPB/C, and were sacrificed on day 80. As depicted in Figure 6c, there were no differences in organ weights (heart, liver, spleen, lung, kidney, and brain) between PBS and Cu‐HPB/C groups. Additionally, Cu‐HPB/C had negligible effects on hematology parameters (Figure 6d), and blood biochemistry parameters (Figure 6e) related to hepatic (ALT, AST, and ALP) and renal (CR and UREA) functions. Besides, histological hematoxylin and eosin (H&E) staining of major organs revealed no morphological changes or damages in Cu‐HPB/C‐treated mice (Figure 6f). In summary, the above results indicate the excellent biosafety of Cu‐HPB/C, permitting its potential for in vivo applications.

### 2.11 Biodistribution of Cu‐HPB/C

**Figure 7:**
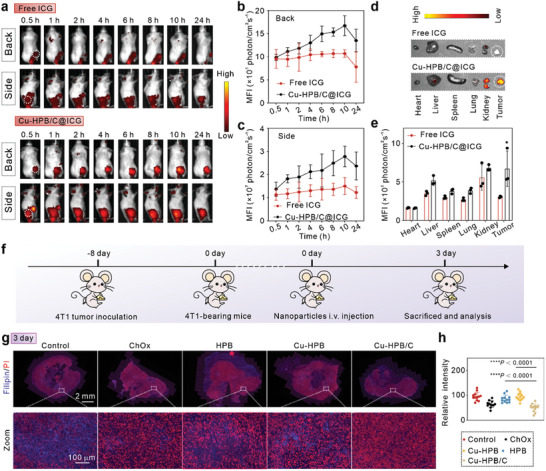
Biodistribution and in vivo cholesterol depletion of Cu‐HPB/C (n = 3). a) Fluorescence images of free ICG and Cu‐HPB/C@ICG distribution in 4T1‐bearing mice at different time points. b,c) Statistical fluorescent intensity of tumor areas of mice from (a). d,e) Fluorescence images of major organs and tumor tissues at 72 h post‐treatment (d) and corresponding statistical results (e). f) Scheme of Cu‐HPB/C for cholesterol depletion detection. g,h) Fluorescence images of tumors from mice receiving various treatments on day 3 (g) and corresponding statistical results (h). Statistical significance is denoted as ^*^ *p* < 0.05, ^****^ *p* < 0.0001, analyzed by one‐way ANOVA, followed by Dunnett's multiple comparisons test. Data represent mean ± s.d.

The tumor accumulation of Cu‐HPB/C was evaluated by an in vivo distribution assay. We synthesized ICG‐labeled Cu‐HPB/C (Cu‐HPB/C@ICG) to trace its biodistribution both in vivo and in situ. The loading efficacy of ICG was calculated to be 100.00% based on the UV–vis spectra standard curve (Figure S23, Supporting Information). Mice bearing 4T1 tumors were divided into two groups, receiving either Cu‐HPB/C@ICG or an equivalent of free ICG. In vivo images (**Figure** **7**a) and analysis of tumoral fluorescence intensity (Figures 7b,c) were conducted using an IVIS system. Cu‐HPB/C@ICG demonstrated significantly higher fluorescence intensity at the tumor site compared to free ICG, indicating excellent tumor accumulation efficiency. 24 h post‐injection, the mice were euthanized, and their tumors and major organs were imaged. As shown in Figures 7d,e, mice treated with Cu‐HPB/C@ICG displayed higher fluorescence intensity at tumor sites than those treated with free ICG, further confirming enhanced tumor targeting and its potential for cholesterol depletion and tumor therapy.

### 2.12 In Vivo Cholesterol Depletion

We then investigated the cholesterol depletion capability of Cu‐HPB/C in 4T1 tumor‐bearing mice (Figure 7f). In this experiment, tumor‐bearing mice received intravenous (i.v.) injections of PBS (control), ChOx, HPB, Cu‐HPB, or Cu‐HOB/C. Tumoral cholesterol levels were assessed using Filipin staining. Three days post‐injection, treatments with ChOx, HPB, and Cu‐HPB showed no significant changes in blue fluorescence compared to the control group. However, Cu‐HPB/C treatment resulted in dimmer blue fluorescence signals (Figures 7g,h), indicating reduced cholesterol levels in Cu‐HPB/C‐treated tumors.

### 2.13 Tumor Inhibition and Antimetastasis of Cu‐HPB/C

**Figure 8:**
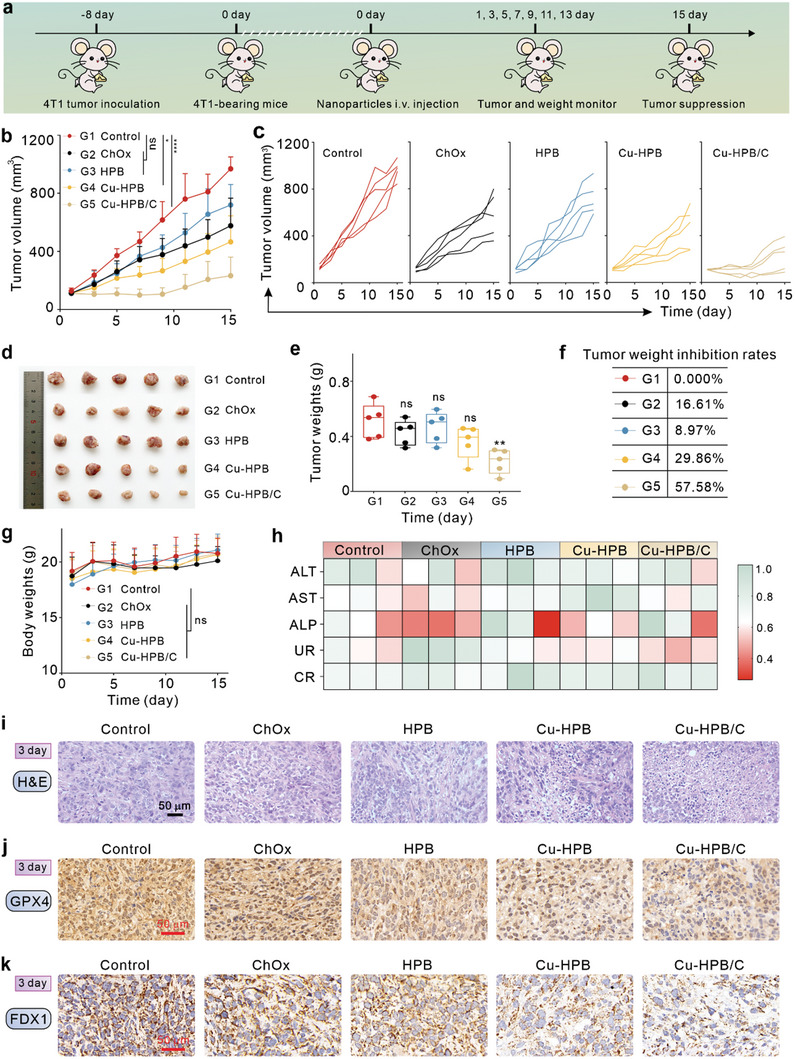
Tumor inhibition in vivo (n = 5). a) Treatment schedule. b,c) Total tumor growth curves (b) and corresponding individual growth curves (c) of 4T1 tumor‐bearing mice receiving various treatments. d,e) Tumor weights (e) and corresponding photos (d) of tumors on day 14. f) Tumor inhibition rates of each group were calculated from (d). g) Body weights of mice during various treatments. h) Biochemical analysis of serum from mice on day 14. i–k) H&E staining (i), GPX4 IHC (j), and FDX1 IHC staining (k) of tumor slices from mice on days 3. Statistical significance denoted as ^**^ *p* < 0.01, ns: not significant, analyzed by one‐way ANOVA, followed by Dunnett's multiple comparisons test. Data represent mean ± s.d.

The effective in vivo cholesterol depletion ability of Cu‐HPB/C inspired us to explore its tumor inhibition and antimetastasis effects. The treatment process is illustrated in **Figure** **8**a. In a typical experiment, twenty‐five 4T1 tumor‐bearing mice female Balb/c mice were divided into five groups (n = 5): control (PBS, G1), ChOx (G2), HPB (G3), Cu‐HPB (G4), and Cu‐HPB/C (G5). The mice received assigned formulations, and their body weight and tumor size were recorded every two days. As shown in Figures 8b,c,f, tumors from mice treated with ChOx and HPB exhibited moderate tumor inhibition with tumor growth inhibition (TGI) ratios of 16.61 and 8.97%, respectively. In contrast, Cu‐HPB demonstrated superior tumor inhibition compared to HPB, attributed to the enhanced catalytic ability conferred by copper doping. Notably, Cu‐HPB/C significantly inhibited tumor growth, with a TGI ratio of 57.58%.

After 14 days of treatment, the mice were euthanized, and their tumors were photographed (Figure 8d) and weighed (Figure 8e). These results indicate that cholesterol depletion by ChOx enhances the therapeutic efficacy of Cu‐HPB. Additionally, mice that received various treatments displayed no significant changes in body weights (Figure 8g) and blood biochemical parameters (Figure 8h), confirming the biocompatibility and biosafety of treatments. Subsequently, we investigated the therapeutic mechanisms of Cu‐HPB/C by performing H&E and immunohistochemical (IHC) staining slices on the tumor after 3 and 14 days of treatment. As shown in Figure 8i and Figure S24a (Supporting Information), Cu‐HPB/C induced severe tumor damage. Furthermore, Cu‐HPB/C significantly suppressed the expression of GPX4 compared to ChOx and Cu‐HPB (Figure 8j; Figure S24b, Supporting Information), indicating the induction of ferroptosis. Additionally, Cu‐HPB suppressed FDX1 expression compared to HPB, and this inhibition was enhanced synergistically with ChOx (Cu‐HPB/C) (Figure 8k; Figure S24c, Supporting Information). These findings suggest that Cu‐HPB/C induces both ferroptosis and cuproptosis and that cholesterol depletion by ChOx enhances the tumor therapeutic efficacy of Cu‐HPB.

**Figure 9:**
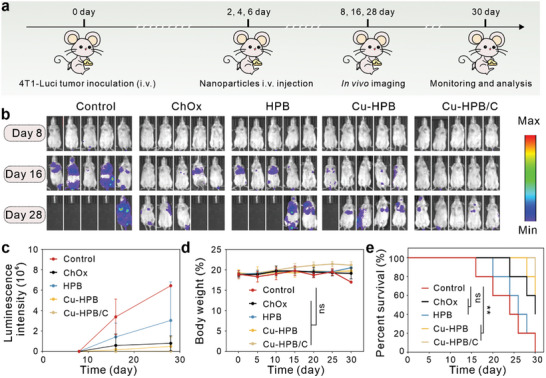
Tumor metastasis inhibition in vivo (n = 5). a) Treatment schedule. b,c) In vivo bioluminescence images (b) and corresponding luminescence intensity (c) of mice after treatment with various formulations. d) Body weights of mice during various treatments. e) Mouse survival ratios of mice during the treatments. Statistical significance denoted as ^**^ *p* < 0.01, ns: not significant, analyzed by one‐way ANOVA, followed by Dunnett's multiple comparisons test. Data represent mean ± s.d.

Next, we investigated the in vivo antimetastasis effects using a 4T1 metastasis model. The treatment process is illustrated in **Figure** **9**a. In this experiment, twenty‐five female Balb/c mice were injected with 5 × 10^5^ luciferase‐expressing 4T1 cells (Luci‐4T1) via the tail vein on day 0. These mice were divided into five groups (n = 5): 1) PBS (control); 2) ChOx; 3) HPB; 4) Cu‐HPB; and 5) Cu‐HPB/C. On days 2, 4, and 6, the mice received intravenous injections according to their assigned treatments. Body weights were recorded every two days. Bioluminescence imaging was performed on days 8, 16, and 28 to visualize tumor metastasis. As shown in Figures 9b,c, the control group exhibited strong bioluminescence signals on day 16 and were close to death, indicating severe tumor metastasis. In contrast, ChOx‐treated mice displayed minimal bioluminescence signals, indicating that ChOx holds the ability to tumor metastasis inhibition. Cu‐HPB treatment showed higher metastasis inhibition efficacy compared to HPB. Notably, Cu‐HPB/C‐treated mice exhibited significantly lower signals on days 16 and 28 compared to the other groups, suggesting notable efficacy in inhibiting tumor metastasis. As can be seen from Figure 9d, Cu‐HPB/C treatment had minimal effects on body weights, demonstrating high biocompatibility. Furthermore, Cu‐HPB/C significantly extended mouse survival (Figure 9e). These findings indicate that the Cu‐HPB/C nano platform effectively inhibits tumor metastasis and prolongs survival in mice.

## 3 Conclusion

In this study, we introduced a novel biocompatible nanoplatform, Cu‐HPB/C, designed for efficient cholesterol depletion and demonstrated its therapeutic efficacy in targeting triple‐negative breast cancer and inhibiting metastasis. The Cu^2+^‐doped hollow Prussian blue (Cu‐HPB) effectively serves as a carrier and enhancer for cholesterol oxidase (ChOx), boosting its stability, tumor‐targeting ability, and enzymatic activity under physiological conditions. Transcriptomic analysis revealed that Cu‐HPB/C modulates various cellular pathways, including cholesterol metabolism, oxidative phosphorylation, the TCA cycle, and ferroptosis, thereby inducing significant changes in gene expression profiles and promoting cancer cell death. The acidity‐triggered release of Cu^2+^ within the tumor microenvironment enhances ChOx activity, leading to substantial cholesterol depletion and disruption of lipid rafts, which impedes cell invasion and migration. Additionally, the production of H_2_O_2_ from the oxidase reaction further augments the chemodynamic therapeutic efficacy of Cu‐HPB, contributing to enhanced oxidative stress and ferroptosis. Our comprehensive in vitro and in vivo experiments underscore the significant antitumor and antimetastatic potential of Cu‐HPB/C, showcasing its ability to induce ferroptosis, cuproptosis, and apoptosis in cancer cells. By effectively targeting and modulating cholesterol metabolism, Cu‐HPB/C offers a promising strategy for overcoming the challenges associated with cholesterol accumulation in cancer cells, thereby providing a robust approach for tumor therapy and metastasis inhibition. This study not only highlights the therapeutic potential of Cu‐HPB/C but also underscores the importance of targeting metabolic vulnerabilities in cancer treatment. The successful implementation of Cu‐HPB/C paves the way for future advancements in nanomedicine aimed at precise and efficient cancer therapy through metabolic modulation.

## 4 Experimental Section

### Materials

K_3_[Fe(CN)_6_]·H_2_O, PVP (K30), and Filipin complex were purchased from Sigma–Aldrich. CuSO_4_, Cholesterol Oxidase (ChOx), wortmannin (WM), cytochalasin D (CD), β‐ cyclodextrin (β‐CD), polyvinyl pyrrolidone (PVP, 40K), and chlorpromazine (CPZ) were obtained from Aladdin. Hydrochloric acid (HCl) and hydrogen peroxide (H_2_O_2_) were sourced from Sinopharm. The Liperfluo kit, Annexin V FITC/PI apoptosis kit, and cell counting kit (CCK‐8) were obtained from Dojindo. The ^•^OH fluorescent kit was bought from BBoxbio. Amplex Red was purchased from Beyotime. Tris (4,7‐diphenyl‐1,10‐phenanthroline) ruthenium (II) dichloride complex (RDPP) was acquired from Macklin. All antibodies used in Western blotting assays were purchased from Proteintech.

### Synthesis of Cu‐HPB/C—Synthesis of PB

0.1 g of K_3_[Fe(CN)_6_]·H_2_O and 3 g of PVP were dissolved in 20 mL of ultrapure water in a round‐bottom flask. The solution was subjected to ultrasound for several minutes, followed by the addition of 17.5 µL of HCl and stirring for 0.5 h. The mixture was then transferred into a reaction vessel and reacted at 80 °C for 20 h, yielding PB nanoparticles. The acidic degradation behavior of PB was assessed by incubating the nanoparticles in buffers at pH 7.4 and 5.5 for 12 and 24 h, with TEM imaging used to evaluate structural changes.

### Synthesis of Cu‐HPB/C—Synthesis of HPB

5 mg of PB and 25 mg of PVP were dissolved in 5 mL of HCL solution in a round‐bottom flask. The solution was subjected to ultrasound for several minutes until completely dissolved. The mixture was then transferred into the reaction vessel and reacted at 140 °C for 4 h to obtain HPB.

### Synthesis of Cu‐HPB/C—Synthesis of Cu‐HPB

5 mg of HPB was dissolved in 10 mL of ultrapure water in a centrifuge tube. Different amounts of CuSO_4_ solution were added to the centrifuge tube to achieve a final concentration of 10, 25, 50, and 100 mm. The mixture was stirred at room temperature for 8 h to produce Cu‐HPB.

### Synthesis of Cu‐HPB/C—Synthesis of Cu‐HPB/C

1 mg of Cu‐HPB was dissolved in 5 mL of ultrapure water in a centrifuge tube. Then, 1 mg of ChOx was added to the above solution and stirred overnight at 4 °C. The mixture was centrifuged several times to remove unloaded ChOx, resulting in Cu‐HPB/C. The ChOx concentration in the supernatant after centrifugation was measured by the BCA Protein Assay kit to evaluate the drug loading efficacy and encapsulation efficiency.

### Cholesterol Depletion by Cu‐HPB/C

The cholesterol depletion rate of Cu‐HPB/C was evaluated in different pH buffer solutions (5.5, 6.5, and 7.4) under hypoxia and oxygen conditions. A reaction mixture containing 50 µL of Cu‐HPB/C (2 mg mL^−1^) and 200 µL of cholesterol (1 mg mL^−1^) was prepared in 5 mL of PBS (pH 5.5, 6.5, and 7.4) under specified oxygen conditions, with free ChOx as a control. After 12 h, the residual cholesterol was quantified using a cholesterol assay kit.

### Cholesterol Oxidase Activity Measurement

The cholesterol oxidase activity was measured by the 4‐amino‐antipyrine colorimetric method. First, the final concentration of 1 mm 4‐amino‐antipyrine, 5 mm phenol, 5 U mL^−1^ peroxidase, and 20 mm sodium phosphate was mixed and the pH was titrated to 7.0 to obtain assay solution. Then, 300 µg cholesterol dissolved in dimethylformamide containing 5% TritonX‐100 was added to 1.0 mL assay solution and pre‐incubated for 3 min. After that, 20 µg mL^−1^ ChOx was incubated with various concentrations of Cu^2+^ (0, 0.01, 0.025, 0.05, 0.1, 0.15, 0.2, 0.5, 1, 2, and 4 µm) and added for reaction. The mixture was boiled in a water bath for 2 min and placed in an ice bath for 2 min. Finally, the 500 nm absorption of the mixture was read in a MultiScan reader. Similar procedures were conducted to evaluate the influence of pH on the enzyme activity of ChOx except for the reaction systems were titrated to different pH instead of adding various concentrations of Cu^2+^.

### Cell Uptake of the Cu‐HPB/C

Murine breast cancer 4T1 cells were infected with the firefly luciferase virus to obtain the firefly luciferase‐expressed 4T1 (Luc‐4T1) cells. The 4T1 cells and Luc‐4T1 cells were cultured in DMEM supplemented with 10% fetal bovine serum and 1% penicillin‐streptomycin and incubated at 37 °C under 5% CO_2_. All cells tested negative for mycoplasma contamination and rodent pathogens.

The intracellular uptake behavior of the Cu‐HPB/C nanoplatforms was analyzed by confocal laser scanning microscope (CLSM, Zeiss, LSM780) and flow cytometry (FCM, Agilent, Novocyte 3130). FITC was used to label the nanoplatforms (Cu‐HPB/C@FITC). Approximately 1 × 10^5^ cells were seeded into confocal dishes and cultured overnight. The cells were then incubated with 50 µg mL^−1^ Cu‐HPB/C@FITC at 37 °C for different time points (0, 1, 2, 4, 8, 12, and 24 h). After incubation, the cells were washed three times with PBS and observed using CLSM. For FCM analysis, the same procedures were followed, and the cells were harvested and quantified by using an FCM, with data analysis performed using FlowJo software.

### Cell Uptake Pathways

To verify the cell uptake pathways of Cu‐HPB/C, 4T1 cells pretreated at 4 °C or with different endocytic inhibitors including, 100 nM WM, 1 µg mL^−1^ Filipin, 10 µg mL^−1^ CPZ, 0.2 µg mL^−1^ CD, and 10 mg mL^−1^ MβCD, at 37 °C for 1 h. subsequently, the cells were washed twice with PBS and incubated with 50 µg mL^−1^ Cu‐HPB/C@FITC for an additional 5 h. The 4T1 cells were then harvested, and their fluorescent intensity was quantified by using FCM.

### Lysosomal Co‐Localization Assay

To evaluate the lysosomal co‐localization of Cu‐HPB/C, 4T1 cells were incubated with 50 µg mL^−1^ of Cu‐HPB/C@FITC for various time points (2, 6, and 10 h). Following incubation, cells were fixed with 4% paraformaldehyde for 10 min, stained with DAPI for nuclear visualization, and labeled with a lysosome‐specific tracker (red) for 30 min to identify lysosomes. Fluorescent images were subsequently captured using a CLSM.

### Cytotoxicity Assay

To assess the cytotoxicity of HPB, Cu‐HPB, and Cu‐HPB/C, 4T1 cells were exposed to HPB, Cu‐HPB, and Cu‐HPB/C with different concentrations (0, 20, 40, 60, 80, and 100 µg mL^−1^) of each compound for 24 h. Cell viability was then measured using the CCK8 assay.

### Calcein‐AM/PI Test

4T1 cells were divided into four groups: 1) PBS, 2) HPB (50 µg mL^−1^), 3) Cu‐HPB (50 µg mL^−1^), and 4) Cu‐HPB/C (50 µg mL^−1^). The cells were incubated with the respective formulations for 24 h, followed by staining with Calcein‐AM/PI for 30 min and imaging using CLSM.

### Cell Colony Formation Assay

4T1 cells were seeded in 6‐well plates at a density of 600 cells per well in complete culture media and treated with different formulations. The media were replaced every three days. After two weeks, the cells were fixed with 4% paraformaldehyde for 20 min, stained with 1 mg mL^−1^ crystal violet for 10 min, and observed under an optical microscope (Olympus, CKX53).

### Transwell Migration Assay

The Transwell assay was employed to evaluate the cell migration following treatment with different formulations. 4T1 cells (200 µL, 1 × 10^5^ mL^−1^) were preconditioned with serum‐free media and cultured in the upper chamber of Transwell (Corning Inc., Corning, NY, USA). Media containing serum and 50 µg mL^−1^ of various probes (HPB, Cu‐HPB, and Cu‐HPB/C) or PBS were added to the lower chamber to induce cell migration. After 24 h of incubation, cells in three randomly selected microscopic fields were pictured and recorded.

### Measurement of Cell Adhesion Ability

For the cell adhesion assay, 96‐well plates were coated with 10 µg mL^−1^ collagen type I overnight. 4T1 cells were suspended in complete media with 50 µg mL^−1^ of various probes (HPB, Cu‐HPB, and Cu‐HPB/C) or PBS and seeded onto the 96‐well plates at a density of 1 × 10^5^/well. The cells were allowed to adhere for 2 h, then washed three times with PBS. Subsequently, the cells were fixed with 4% paraformaldehyde and stained with 1 mg mL^−1^ crystal violet for 10 min. Finally, the attached cells were lysed with 30% glacial acetic acid for 15 min, and the absorbance at 570 nm was recorded.

### Measurement of Cell Invasion Ability

Matrigel (Corning Inc., Corning, NY, USA) was diluted with PBS to a concentration of a least 3 mg mL^−1^ and added to the upper Transwell chamber, then incubated for 30 min at 37 °C. After removing the liquid from the upper chamber, 4T1 cells (200 µL, 1 × 10^5^ mL^−1^) were preconditioned with serum‐free media and cultured in the upper chamber. Media containing serum and 50 µg mL^−1^ of various probes (HPB, Cu‐HPB, and Cu‐HPB/C) or PBS were added to the lower chamber to induce cell invasion. After 24 h of incubation, cells in three randomly selected microscopic fields were pictured and recorded.

### Intracellular Cholesterol Content, Lipid Peroxide, and ^·^OH Imaging

Intracellular cholesterol, lipid peroxide, and ^·^OH levels were assessed using the fluorescent probes of Filipin, Liperfluo, and BBoxiProbeO26, respectively. 4T1 cells were seeded in confocal dishes and incubated with 50 µg mL^−1^ of various probes (HPB, Cu‐HPB, and Cu‐HPB/C) or PBS for 24 h. After washing with PBS three times, the cells were labeled with the appropriate probes, and nuclei were stained with DAPI. Fluorescent images were then acquired using CLSM. The fluorescent intensity was calculated using Image J software.

### Western Blot Analysis

4T1 cells were treated with 50 µg mL^−1^ of various probes (HPB, Cu‐HPB, and Cu‐HPB/C) or PBS for 24 h. Cell lysate (RIPA: protease inhibitor: phosphorylated protease inhibitor = 100: 1: 1) was added to the dishes for 30 min to extract cellular proteins. The proteins were then sonicated and centrifuged at 14,000 rpm for 15 min at 4 °C. The supernatant was collected, and protein concentration was determined using a BCA protein assay kit. Protein samples were mixed with 5× loading buffer, boiled at 100 °C for 10 min, separated by 10% or 12.5% SDS‐PAGE, and transferred onto a PVDF membrane. The membrane was blocked with 5% skim milk at room temperature for 2 h and incubated with a primary antibody (1:1000, Proteintech) overnight at 4 °C. After washing three times with TBST for 5 min each, the membrane was incubated with a Goat anti‐Rabbit/Mouse IgG (H+L)‐HRP conjugate (1: 10000, Proteintech) for 1 h. Finally, the PVDF membrane was scanned in a gel imager and the chemiluminescent images were obtained and analyzed using image J for grayscale values. All the western blotting images were used directly without adjusting the brightness.

### Apoptosis Analysis

Cell apoptosis was evaluated using an Annexin V‐FITC/PI apoptosis kit. Briefly, 4T1 cells were seeded in 6‐well plates at a density of 1 × 10^6^ cells per well and incubated overnight. The cells were then treated with 50 µg mL^−1^ of various probes (HPB, Cu‐HPB, and Cu‐HPB/C) or PBS for 24 h. After washing with PBS, the cells were incubated with Annexin/PI reagent in the dark for 15 min at 25 °C. The fluorescent intensity of cells was immediately measured using FCM.

### Hemolysis Assay

All the mice experiments were conducted following protocols approved by the Animal Experimental Ethics Committee of the Fujian Normal University (Approval No. IACUC‐202220006). Female Balb/c mice (4 weeks) were obtained from Beijing Hua Fu Kang Biotechnology Co. Ltd., and the hemolytic assay for Cu‐HPB/C was performed using fresh blood. Triton‐100 and PBS were used as positive and negative controls, respectively. Different concentrations of Cu‐HPB/C were mixed with red blood cells and incubated at 37 °C for 1 h. The absorbance of the supernatant at 576 nm was measured post‐centrifugation. The hemolysis rate (%) was calculated using the following formula: hemolysis rate (%) = [(absorbance value of the test group−absorbance value of the negative control group)/(absorbance value of the positive control group−absorbance value of the negative control group)] × 100%.

### In Vivo Cholesterol Depletion and Therapeutic Efficacy Evaluation

To establish 4T1 tumor‐bearing mice, 2.5 × 10^6^ 4T1 cells in 100 µL of PBS were subcutaneously injected. When tumor volume reached ≈100 mm^3^, the mice received an intravenous injection of different formulations (n = 3): 1) control; 2) ChOx; 3) HPB; 4) Cu‐HPB; and 5) Cu‐HPB/C (180 µg dosage of ChOx per mouse, 200 µg dosage of HPB, Cu‐HPB, and Cu‐HPB/C per mouse). On days 3 and 14, the mice were sacrificed, and tumors were collected and sliced, tumor slices on day 3 were stained with Filipin to assess in vivo cholesterol depletion. Tumor slices on day 3 and day 14 were analyzed using H&E staining, GPX4, and FDX1 immunohistochemical staining to evaluate the therapeutic efficacy of Cu‐HPB/C.

### In Vivo Tumor Inhibition Evaluation

4T1 tumor‐bearing mice were randomly divided into five groups (n = 5): 1) control; 2) ChOx; 3) HPB; 4) Cu‐HPB; 5) Cu‐HPB/C. Each group receives an intravenous injection of the respective schemes (180 µg dosage of ChOx per mouse, 200 µg dosage of HPB, Cu‐HPB, and Cu‐HPB/C per mouse). Tumor volume and body weights of mice were recorded every 2 days. Tumor volume was calculated using the formula:

(1) $$V\left(\mathrm{volume}\right)=\mathrm{length}\times {\mathrm{width}}^{2}/2$$

After 14 days, the mice were euthanized, and their tumors were collected and sectioned for H&E staining and IHC staining. Blood samples were also taken for serum biochemical analysis.

### In Vivo Tumor Metastasis Inhibition

Female Balb/c mice (4 weeks) were randomly divided into five groups (n = 5): 1) control; 2) ChOx; 3) HPB; 4) Cu‐HPB; 5) Cu‐HPB/C. On day 0, the mice were intravenously injected with 5 × 10^5^ Luc‐4T1 cells. Subsequently, on days 2, 4, and 6, the mice received intravenous injections of the assigned formulations (180 µg dosage of ChOx per mouse, 200 µg dosage of HPB, Cu‐HPB, and Cu‐HPB/C per mouse). Body weights were recorded every two days. Bioluminescence imaging was performed on days 8, 16, and 28 to monitor tumor metastasis.

### Long‐Term Toxicity Assay

To evaluate long‐term toxicity, healthy mice were intravenously injected with 20 mg kg^−1^ Cu‐HPB/C (n = 5). After 80 days, the mice were euthanized, and the weight of major organs (heart, liver, spleen, lung, kidney, and brain) was recorded. These organs were then excised for H&E staining. Additionally, whole blood and serum samples were collected for biosafety evaluation. Blood components were counted and compared, and serum levels of aspartate aminotransferase (AST), alanine aminotransferase (ALT), alkaline phosphatase (ALP), creatinine (CRE), and urea (UR) were measured to assess the liver and kidney function.

### Statistical Analysis

Statistical analysis was performed using GraphPad Prism (9.0) and evaluated by analysis of variance (ANOVA). P values of 0.05 or less were considered statistically significant.

## Conflict of Interest

The authors declare no conflict of interest.

## Supporting information



Supporting Information

## Data Availability

The data that support the findings of this study are available from the corresponding author upon reasonable request.
