# Supplementary material for: Cupric Doping Hollow Prussian Blue Nanoplatform for Enhanced Cholesterol Depletion: a Promising Strategy for Breast Cancer Therapy and Metastasis Inhibition
Source: Adv Sci (Weinh). 2024 Nov 28;12(3):2409967. doi: 10.1002/advs.202409967 (PMC11744725; doi:10.1002/advs.202409967)
Supplement: Supplementary file 1 — Supporting Information [file ADVS-12-2409967-s001.docx]

Supporting Information

Cupric Doping Hollow Prussian Blue Nanoplatform for Enhanced Cholesterol Depletion: A Promising Strategy for Breast Cancer Therapy and Metastasis Inhibition

*Shuangqian Yan^†^, Panpan Xue^†^, Ying Sun^†^, Tingjie Bai, Sijie Shao, Xuemei Zeng^*^*


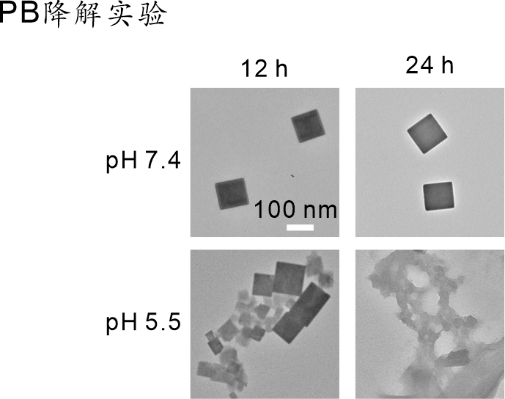
**Figure S1.** The TEM images of PB dissolved in PBS at pH 7.4 and 5.5 storage for 12 h and 24 h. Scale bar is 100 nm.


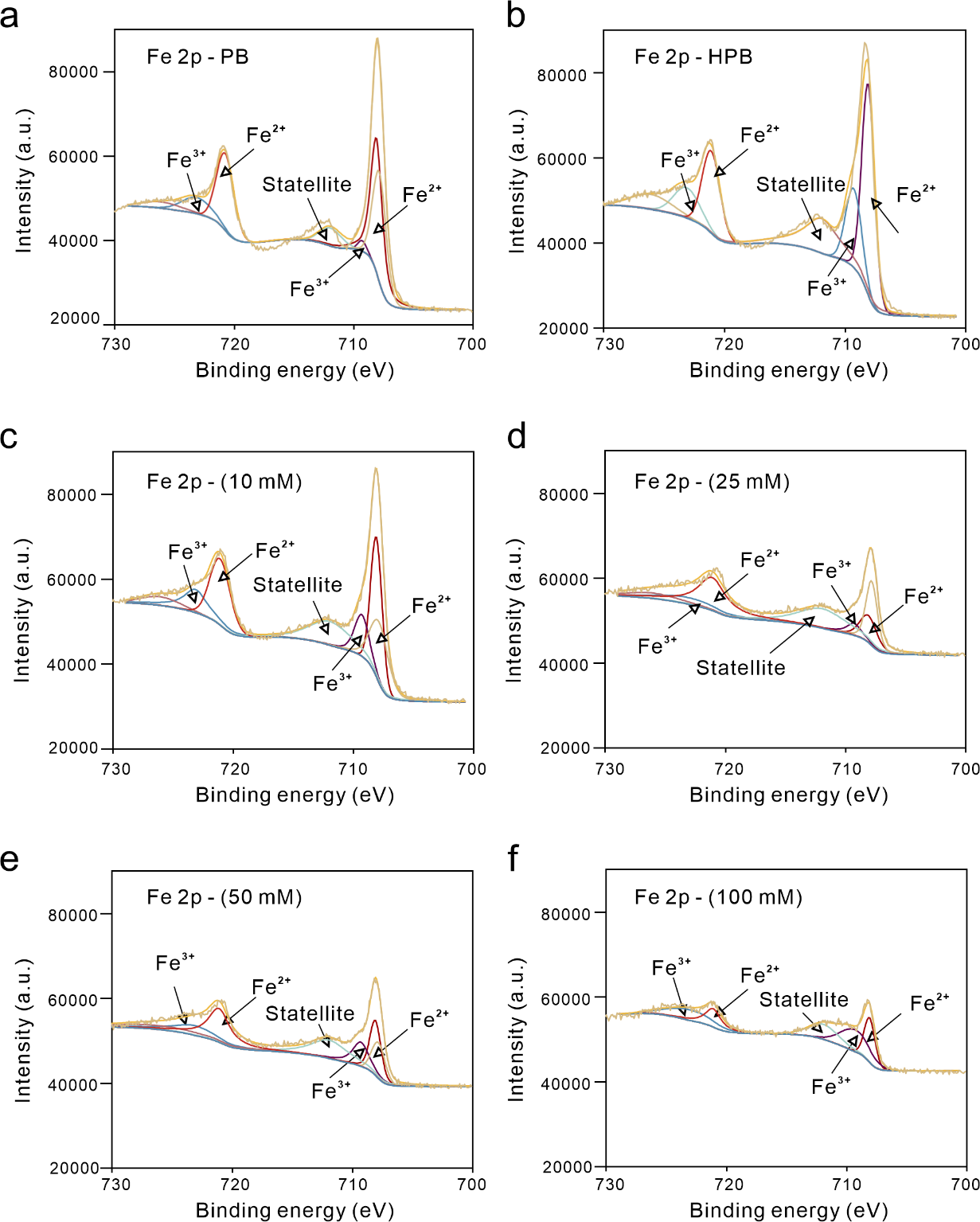
**Figure S2**. (a, b) Fe 2p spectra of PB (a) and HPB (b). (c-f) Fe 2p spectra of HPB following incubating CuSO_4_ with concentrations of 10 mM (c), 25 mM (d), 50 mM (e), and 100 mM (f).


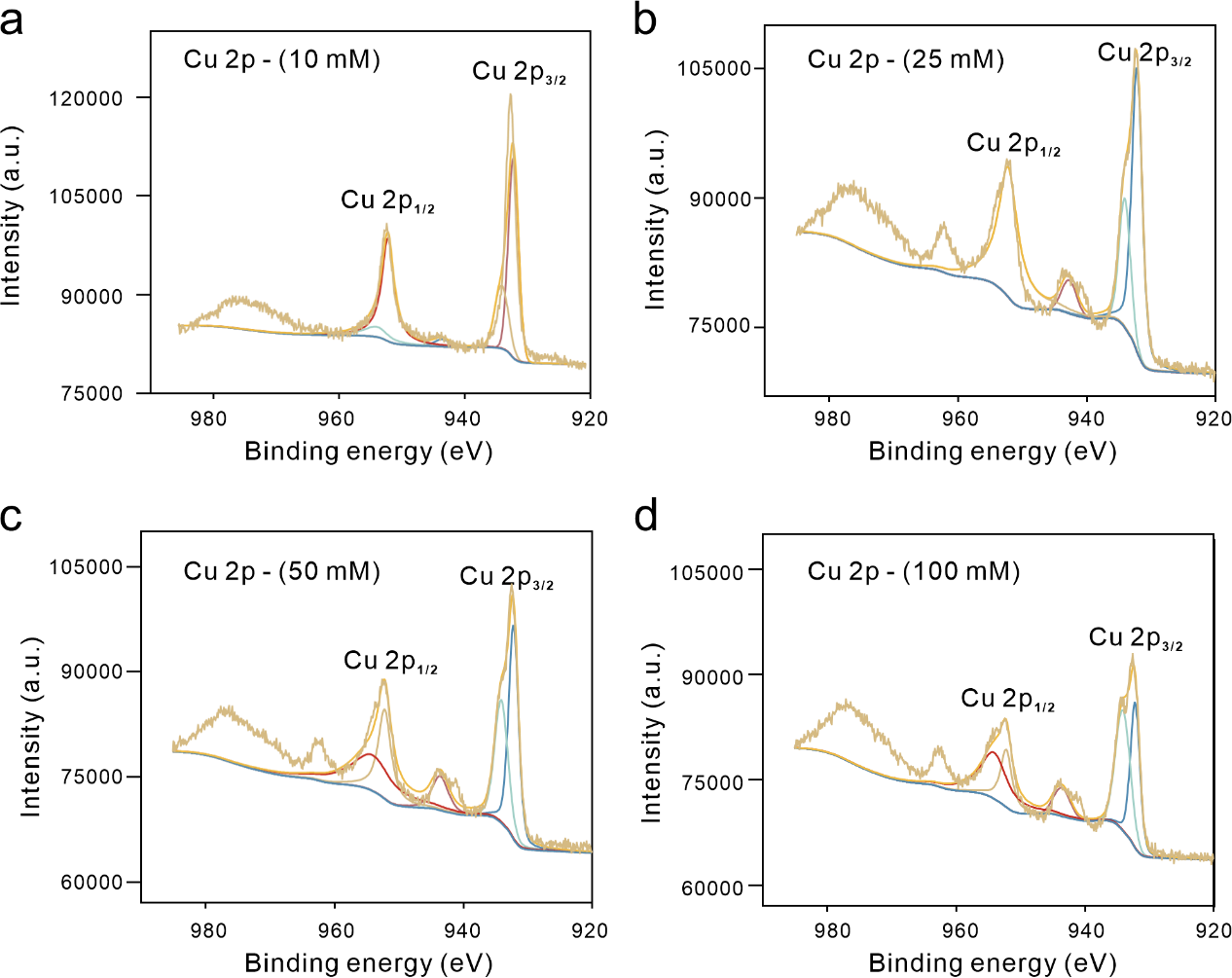


**Figure S3**. (a-d) Cu 2p spectra of HPB following incubating CuSO_4_ with concentrations of 10 mM (a), 25 mM (b), 50 mM (c), and 100 mM (d).


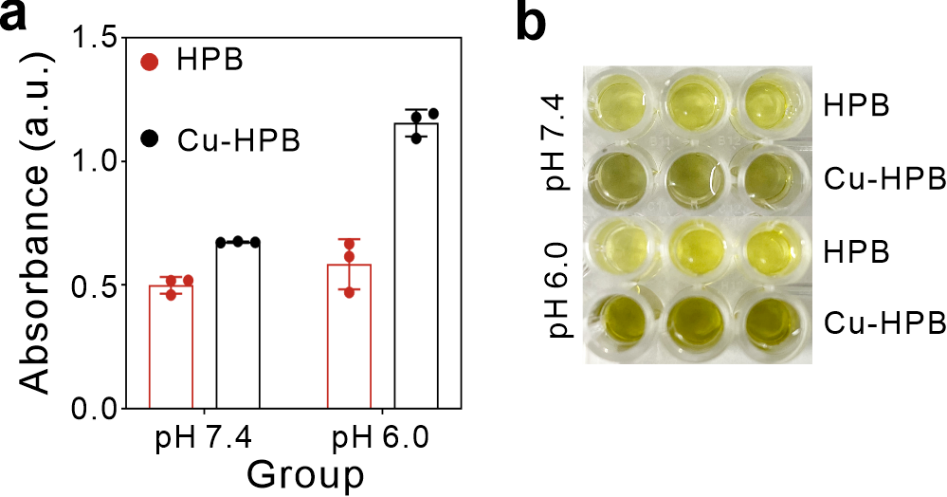


**Figure S4**. (a, b) Absorbance of OPD solutions following treatment with HPB and Cu-HPB at pH 7.4 and 6.0 in the presence of 25 mM H_2_O_2_ (n = 3).


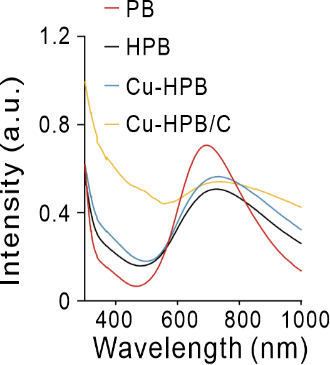
**Figure S5**. UV-vis-NIR spectra of Cu-HPB with various modifications.


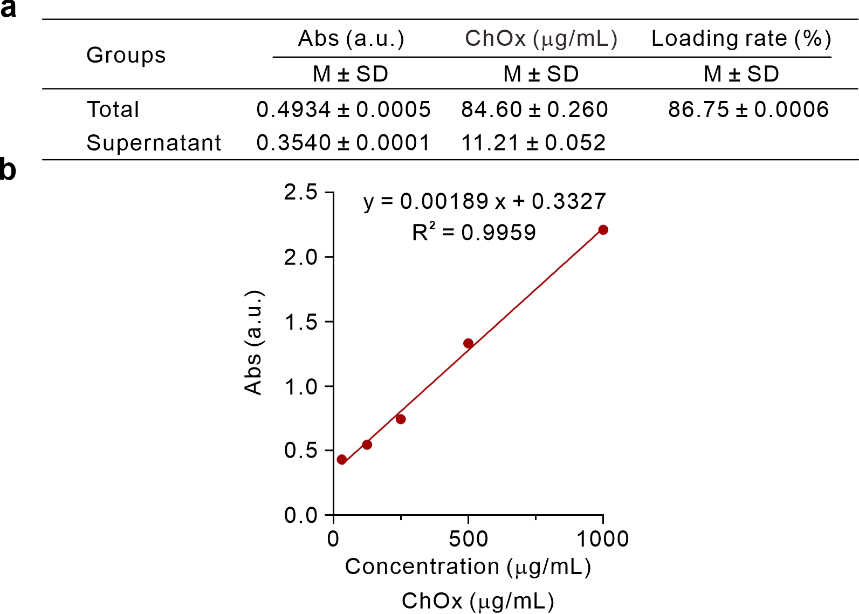
**Figure S6**. (a) The protein contents of total ChOx solution and the supernatant determined by the BCA Protein Assay Kit, and the loading rate. (b) Standard curve of ChOx (n = 3).


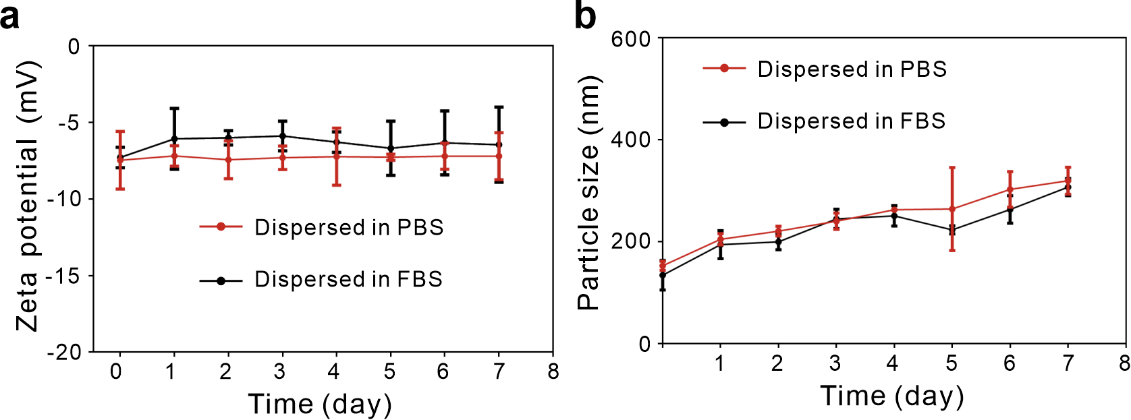


**Figure S7**. (a, b) Changes in Zeta potential (a) and hydrodynamic size of Cu-HPB/C in PBS and DMEM medium with 10% FBS within 7 days (n = 5).


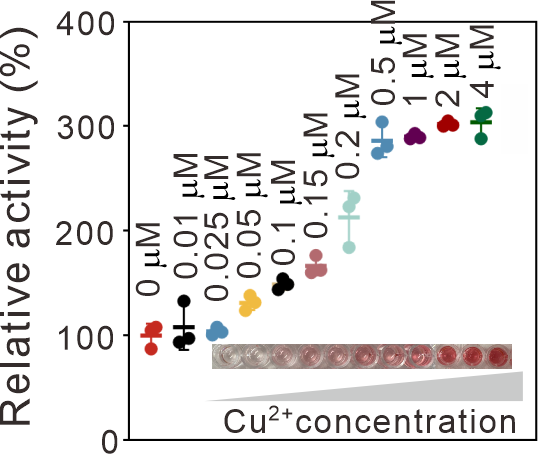


**Figure S8**. Relative activity of ChOx in the presence of various concentration of Cu^2+^ (n = 3).

**
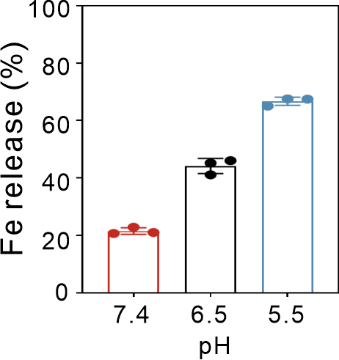
Figure S9.** The Fe release behaviors of Cu-HPB/C under various pH values (n = 3).


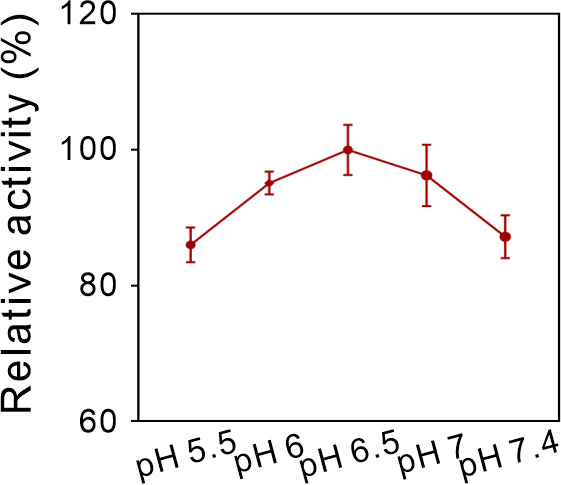


**Figure S10**. Relative activity of ChOx in buffer with different pH values (n = 3).


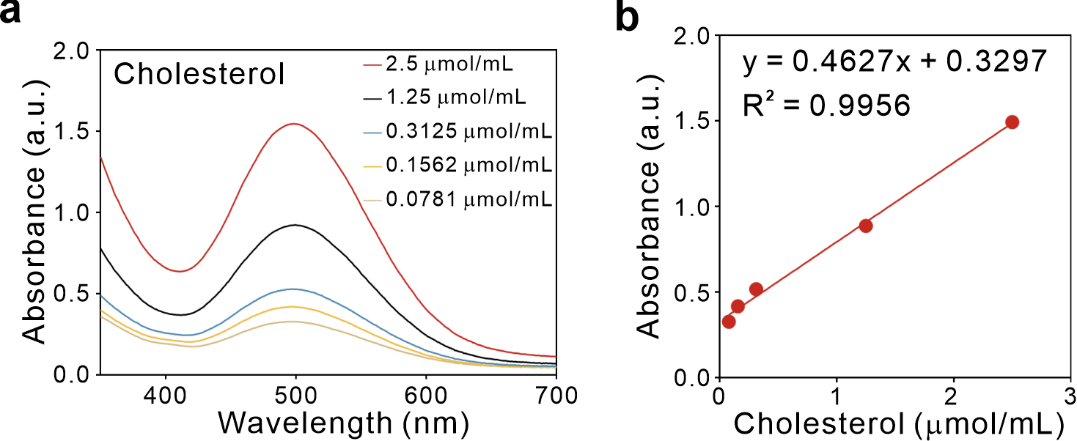


**Figure S11**. (a) UV-vis absorbance of cholesterol with various concentration. (b) Standard curve of cholesterol.


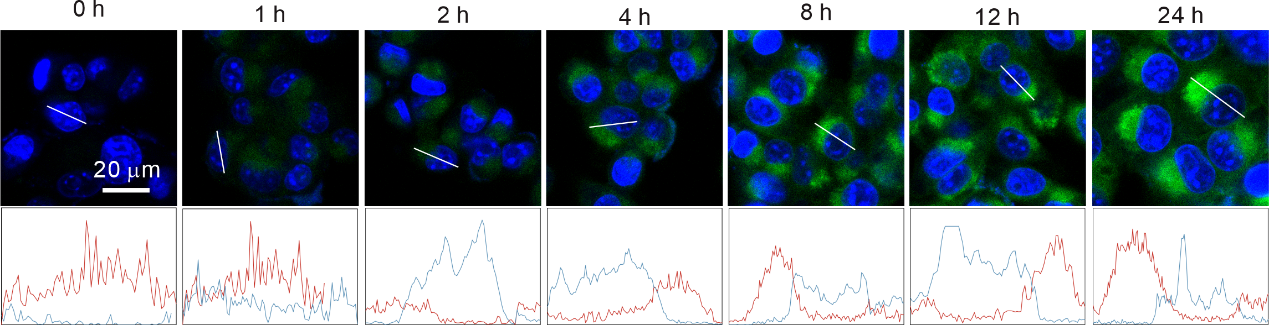


**Figure S12**. Confocal images of 4T1 cells treated with FITC-labeled Cu-HPB/C for various times.


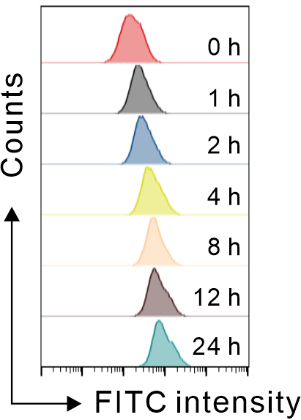
**Figure S13**. Flow cytometry analysis of 4T1 cells treated with FITC-labeled Cu-HPB/C for various times.


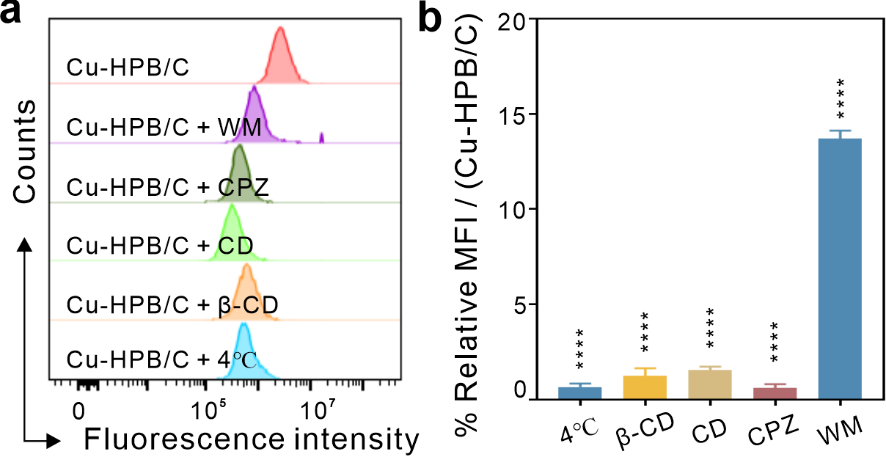


**Figure S14**. (a, b) Fluorescence intensity (a) and corresponding relative MFI (b) in FITC-labeled Cu-HPB/C-treated 4T1 cells treatment with different formulations. n = 3. Statistical significance denoted as ****P < 0.0001, analyzed by one-way ANOVA, followed by Dunnett’s multiple comparisons test. Data represent mean ± s.d.

**Figure S15**. The fluorescent images of lysosome co-localization of Cu-HPB/C in 4T1 cells at indicated time points. The nucleus and lysosome are stained with DAPI, and lysosome tracker, respectively. Scale bar is 20 μm.
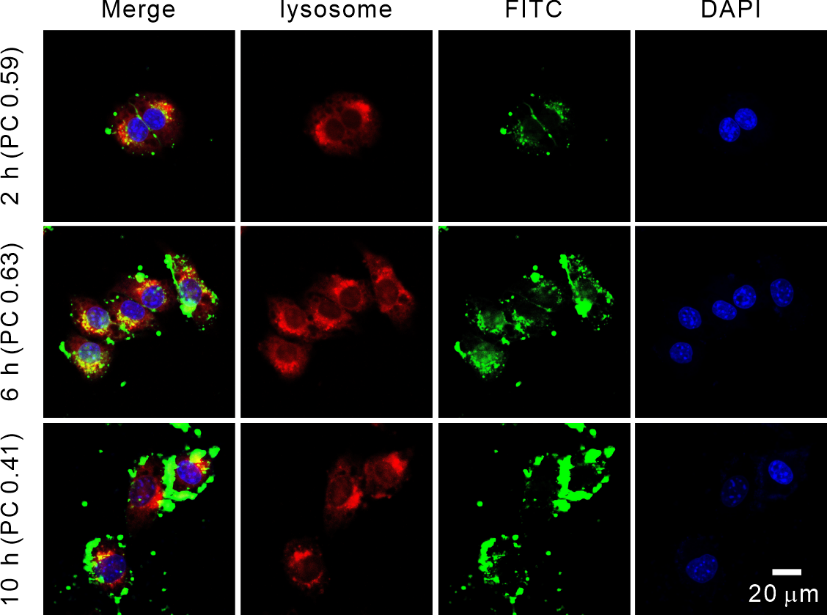


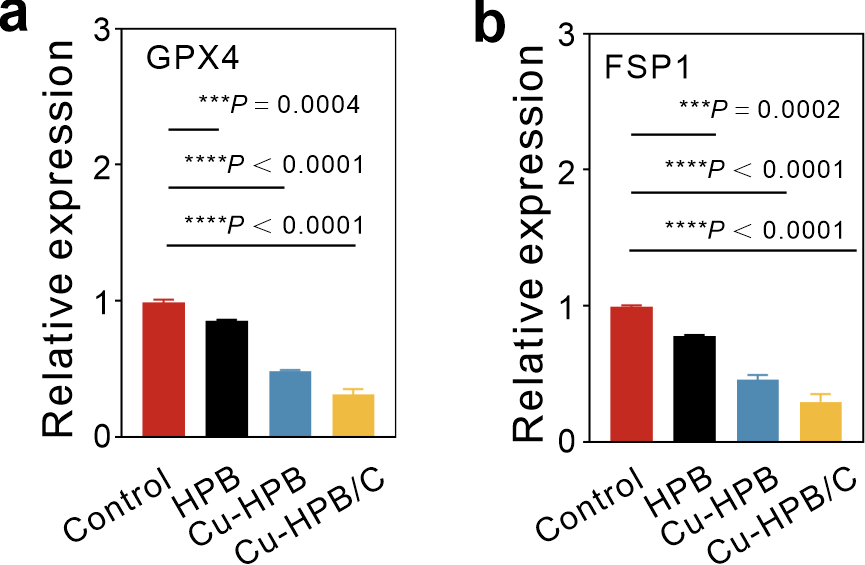


**Figure S16**. (a, b) Relative expression of GPX4 (a) and FSP1 (b) of 4T1 cells by western blot assay. Statistical significance denoted as ***P < 0.001, ****P < 0.0001, analyzed by one-way ANOVA, followed by Dunnett’s multiple comparisons test. Data represent mean ± s.d.


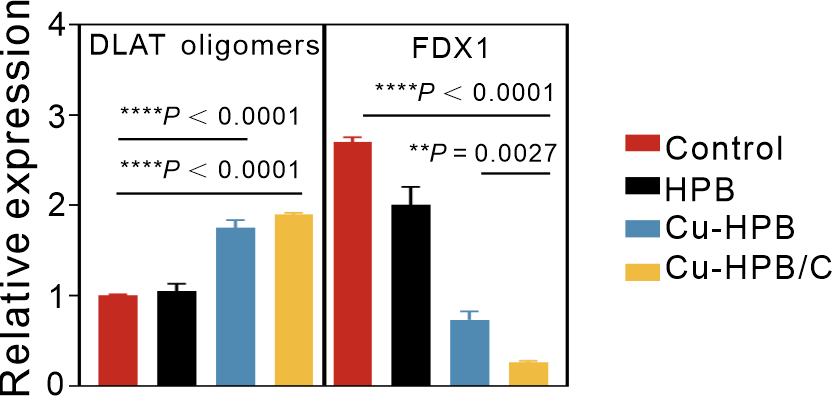


**Figure S17**. Relative expression of DLAT oligomers and FDX1 of 4T1 cells by western blot assay. Statistical significance denoted as **P < 0.01, ****P < 0.0001, analyzed by one-way ANOVA, followed by Dunnett’s multiple comparisons test. Data represent mean ± s.d.


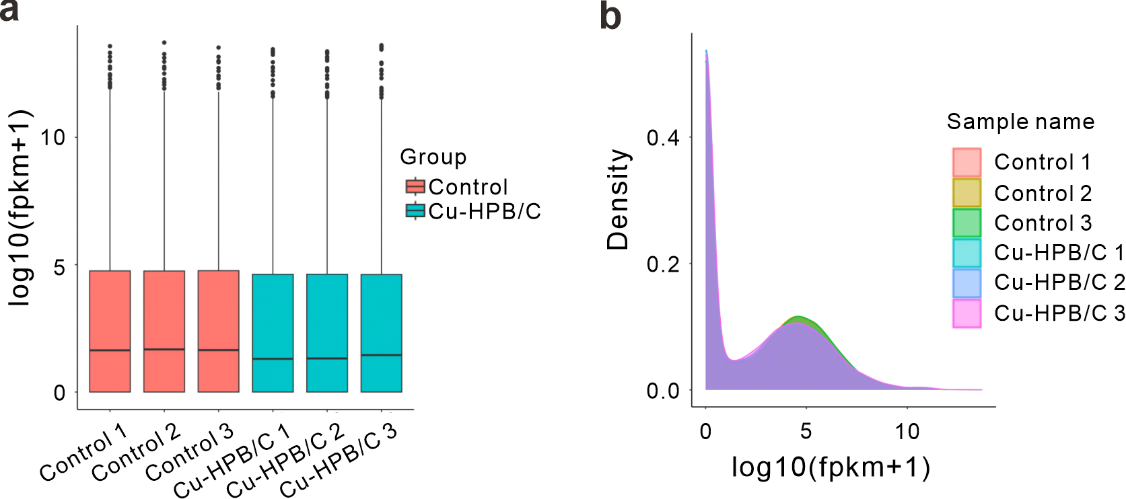


**Figure S18**. (a, b) Correlation between samples from the control and Cu-HPB/C groups.

**
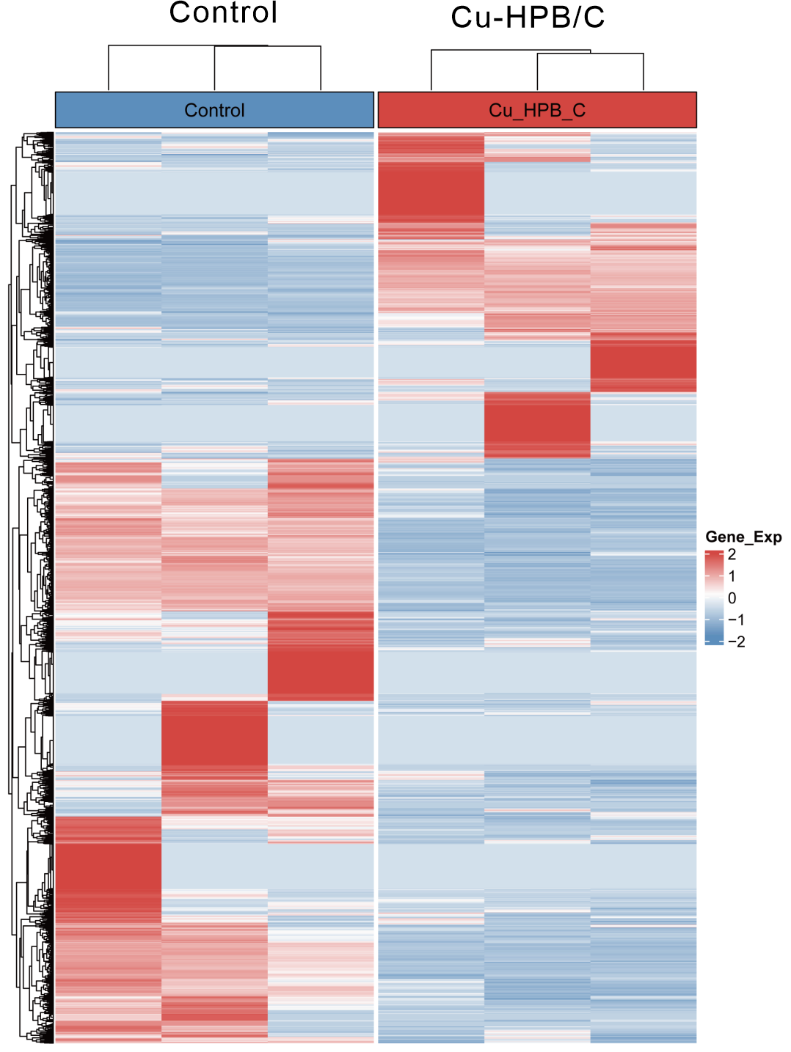
**

**Figure S19**. Heat map of DEGs between the control and Cu-HPB/C groups.


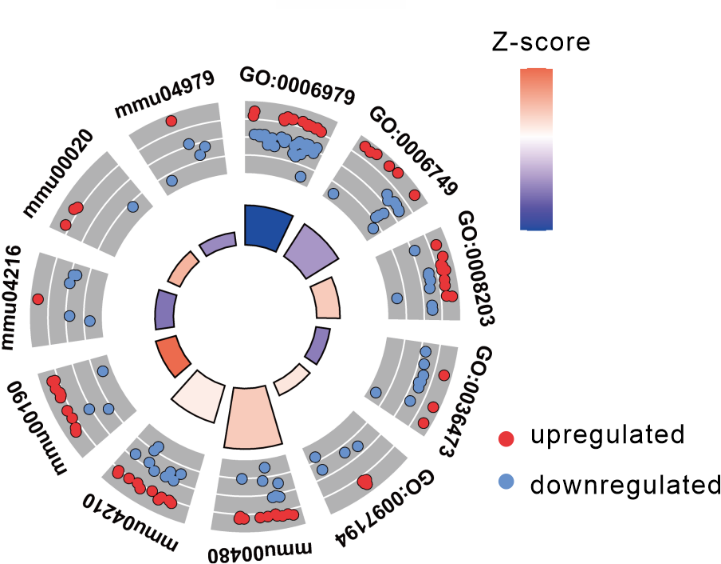


**Figure S20**. GO enrichment analysis of DEGs in the Cu-HPB/C group.


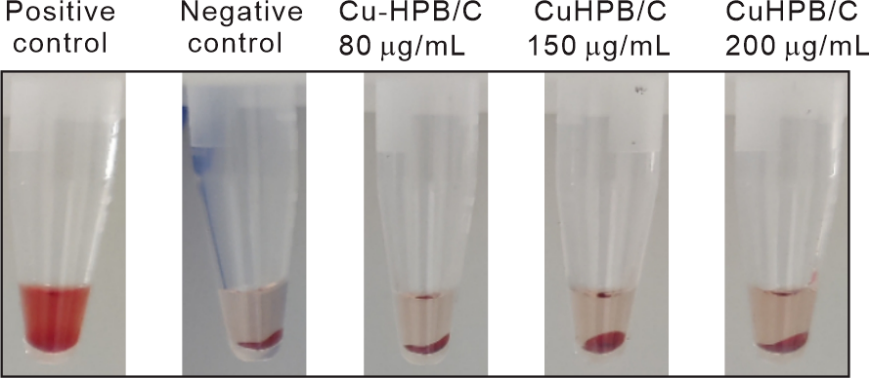


**Figure S21**. Representative photos of red blood cell solutions after incubation with various concentration of Cu-HPB/C, with Triton-X 100 and PBS serving as positive and negative controls, respectively.


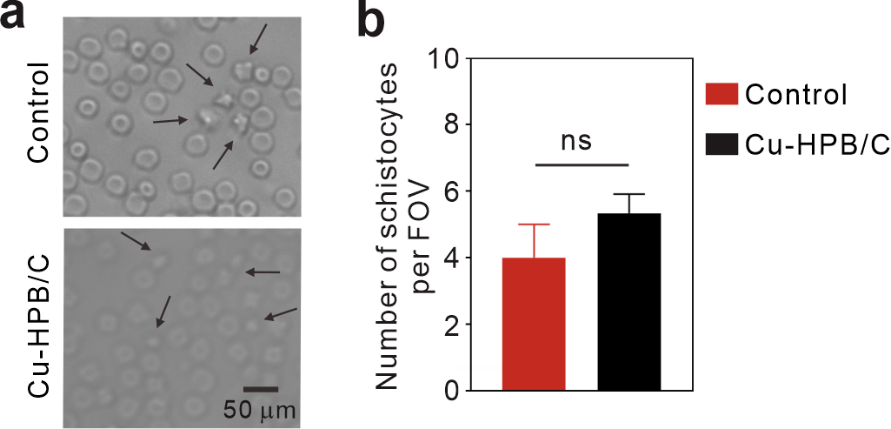


**Figure S22**. (a, b) Representative images (a) and number of schistocytes per fields of view. n = 3. Statistical significance denoted as ns: not significant, analyzed by one-way ANOVA, followed by Dunnett’s multiple comparisons test. Data represent mean ± s.d.


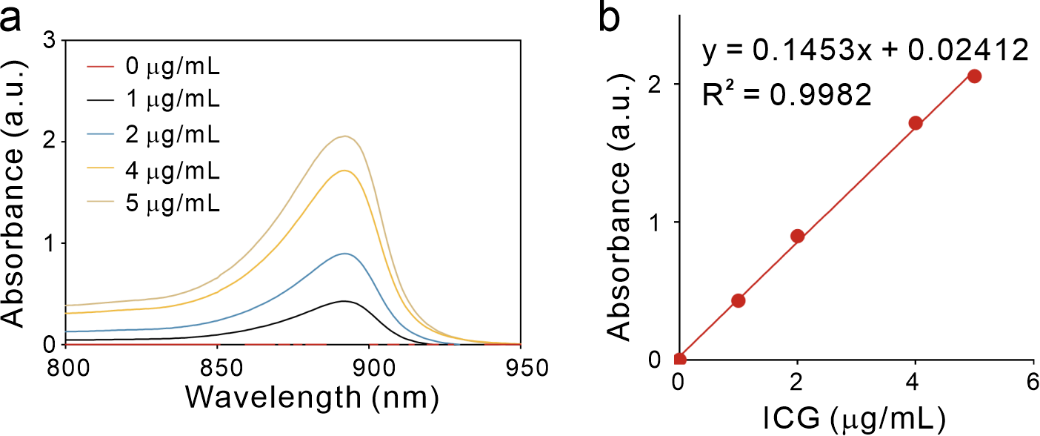


**Figure S23**. (a, b) UV-vis absorbance of ICG with various concentration (0, 1, 2, 4, and 5 μg/mL) (a) and corresponding standard curve (b).


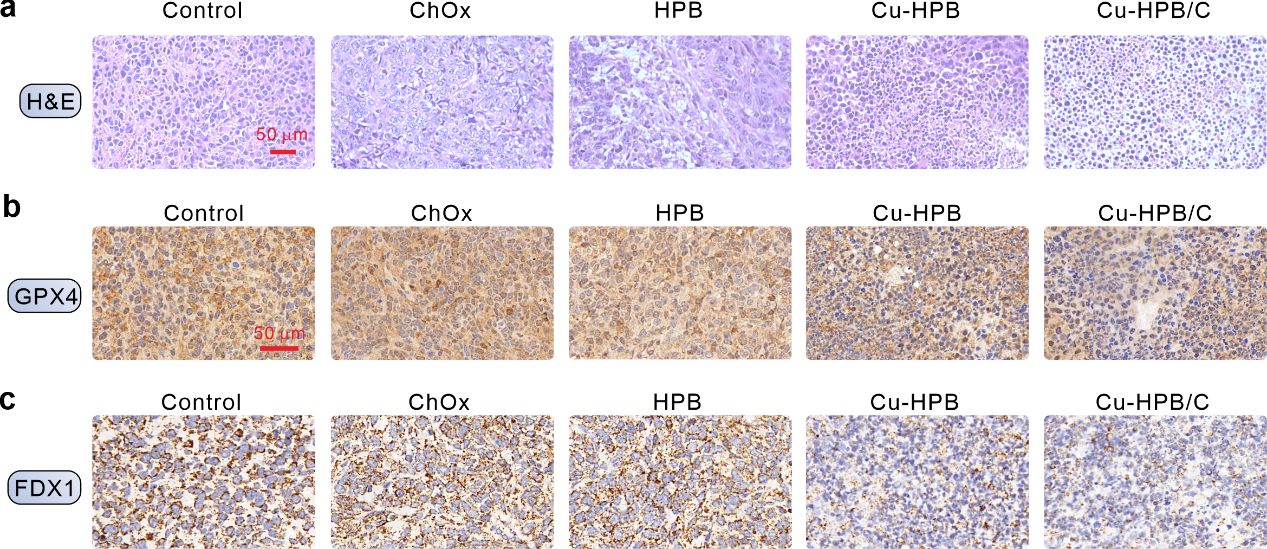
**Figure S24**. (a-c) H&E staining (a), GPX4 IHC (b), and FDX1 IHC staining (c) of tumor slices from mice on days 14.
